# Skin-resident Langerhans cells drive neuropathic pain via chemokine-dependent neuron-immune communication

**DOI:** 10.1172/JCI192328

**Published:** 2026-04-30

**Authors:** Paola Pacifico, Dale George, Nirupa D. Jayaraj, Dongjun Ren, James S. Coy-Dibley, Abdelhak A. Belmadani, Sofia Veronesi, Mirna Andelic, Daniele Cartelli, Grazia Devigili, Raffaella Lombardi, Giuseppe Lauria Pinter, Amy S. Paller, Richard J. Miller, Daniela M. Menichella

**Affiliations:** 1 Department of Neurology, Feinberg School of Medicine, Northwestern University, Chicago, Illinois, USA.; 2 SonoThera Inc., South San Francisco, California, USA.; 3 Department of Pharmacology, Feinberg School of Medicine, Northwestern University, Chicago, Illinois, USA.; 4 Neuroalgology Unit, Fondazione IRCCS Istituto Neurologico Carlo Besta, Milan, Italy.; 5 Department of Medical Biotechnology and Translational Medicine, University of Milan, Milan, Italy.; 6 Department of Dermatology, Feinberg School of Medicine, Northwestern University, Chicago, Illinois, USA.

**Keywords:** Dermatology, Immunology, Neuroscience, Neurodegeneration, Pain, Skin

## Abstract

Neuropathic pain affects over 20 million people in the United States, and painful diabetic neuropathy (PDN), a common complication of diabetes, is among its most prevalent and treatment-resistant forms. Although PDN is characterized by nociceptor dysfunction, the upstream peripheral mechanisms remain incompletely understood. While dorsal root ganglion (DRG) nociceptor hyperexcitability is a hallmark of PDN, emerging evidence suggests that nonneuronal skin cells may modulate nociceptor function. Here, we investigated whether epidermal Langerhans cells (LCs) contribute to neuropathic pain in PDN through neuroimmune signaling. Using a clinically relevant high-fat diet (HFD) mouse model, transgenic LC ablation, behavioral assays, human skin biopsies, and single-cell RNA seq of epidermis and DRG, we found that LC density increased in male diabetic mice in parallel with mechanical allodynia. In skin samples of people with PDN, LCs exhibited increased volume and dendritic complexity correlating with diabetes duration. Genetic depletion of LCs prevented mechanical allodynia and spontaneous pain-like behavior in male, but not female, HFD mice, revealing a sex-dependent contribution. Single-cell and interactome analyses identified male-specific inflammatory LC programs, including upregulation of chemokine signaling pathways. Consistently, LC secretome profiling showed increased CCL2 release, and local CCR2 blockade reversed allodynia. These findings identify epidermal LCs as peripheral regulators of PDN pain and highlight sex-dependent chemokine-mediated neuron-immune communication at the skin-nerve interface.

## Abstract 2

We describe the sex-specific role of Langerhans cells in the epidermis in neuropathic pain development in a mouse model of Painful Diabetic Neuropathy.

## Introduction

Painful diabetic neuropathy (PDN) is a debilitating complication of diabetes characterized by persistent neuropathic pain arising from hyperexcitability of nociceptive neurons in the dorsal root ganglia (DRG) (1, 2). PDN is closely associated with small fiber neuropathy, the earliest pathological hallmark of the disease, which involves both degeneration and aberrant regeneration of DRG axons innervating the skin (3–5). In healthy individuals, these sensory axons extend through cutaneous nerves and terminate within the epidermis, the outermost stratified layer of the skin (6, 7). In PDN, however, cutaneous innervation undergoes extensive remodeling, marked by simultaneous axonal loss and regrowth (3, 8, 9). Despite the clinical importance of this process, the molecular mechanisms that drive nociceptor hyperexcitability and small fiber pathology in PDN remain poorly understood, representing a major barrier to the development of effective therapies.

Sensory axons in the skin do not operate in isolation but form close functional associations with nonneuronal epidermal cells. Axons from distinct nociceptor subpopulations terminate in the epidermis and establish gap junctions and synapse-like contacts with surrounding cells (10–13). While keratinocytes constitute the majority of epidermal cells, the epidermis also contains immune cells, including Langerhans cells (LCs), and specialized T lymphocyte populations that serve as the first line of defense against tissue damage (14–17). An increasing body of evidence implicates these nonneuronal skin cells in the pathogenesis of PDN, with inflammatory processes emerging as key contributors (18–20). Indeed, elevated circulating levels of inflammatory mediators such as IL-6, IL-2, and tumor necrosis factor-α (TNFA), along with increased dermal macrophage density, have been documented in patients with painful — as opposed to nonpainful — diabetic neuropathy (21–24). These findings raise the possibility that local neuro-immune interactions in the skin may critically influence nociceptor dysfunction in PDN.

LCs are specialized epidermal immune cells that play a central role in innate immune surveillance (25–27). Ontogenetically related to macrophages, LCs continuously extend and retract dendritic processes to monitor the local tissue environment and function as resident antigen-presenting cells (26–29). Beyond immune surveillance, LCs secrete and respond to a wide range of inflammatory mediators (18, 21, 30–32), suggesting that they could actively shape local inflammatory states. While LCs have been implicated in several inflammatory skin disorders, their role in neuropathic pain, and PDN in particular, remains largely unexplored. Importantly, LC activation is associated with the release of proinflammatory cytokines capable of sensitizing nociceptor terminals (33, 34), and LC density is increased both in patients with small fiber neuropathy and in rodent models of type 2 diabetes (33, 35). Despite these converging observations, whether and how LCs directly contribute to pain generation in PDN remains unresolved.

Emerging work suggests that epidermal immune cells and sensory afferents engage in extensive bidirectional crosstalk (7). LCs have been shown to physically interact with specific nociceptive terminal afferents, including Mas-related G protein–coupled receptor d–expressing (Mrgprd-expressing) fibers (34, 36). Notably, prior studies have demonstrated that Mrgprd-positive afferents regulate LC recruitment in the skin (36). These findings point to a potentially critical neuro-immune axis linking LCs and nociceptors that may influence neuronal sensitization. However, the molecular mechanisms that promote LC-nociceptor interactions — and the functional consequences of this communication in diabetic neuropathic pain — are currently unknown. Defining these mechanisms represents a key gap in our understanding of PDN pathophysiology.

In these studies, we have addressed this gap in our knowledge by demonstrating a previously unrecognized, dimorphic role for LCs in the development of mechanical allodynia and spontaneous pain in a high-fat diet (HFD) mouse model of PDN. Using integrative interactome analyses of epidermal and DRG single-cell RNA-seq datasets from both male and female mice, we have shown that HFD exposure in PDN males induces alterations in antigen-presenting, inflammatory, and neuro-immune signaling pathways in LCs, suggesting a remodeling of their communication with nociceptive terminal afferents. This remodeling includes upregulation of axon-guidance receptors *Plxnb2* and *Plxna1*, along with chemokine-related and immune signaling genes. Complementary cytokine profiling identified a panel of inflammatory mediators secreted by LCs, with a pronounced increase in chemokine MCP-1/CCL2. Critically, local pharmacological blockade of the CCL22-CCR2 signaling axis reduced mechanical allodynia. Together, these findings establish LCs as active regulators of neuropathic pain in PDN via a defined chemokine-mediated neuro-immune mechanism, revealing an accessible, previously unexplored therapeutic target for alleviating diabetic neuropathic pain.

## Results

### Epidermal LC expansion correlates with mechanical allodynia in a mouse model of painful diabetic neuropathy.

**Figure 1:**
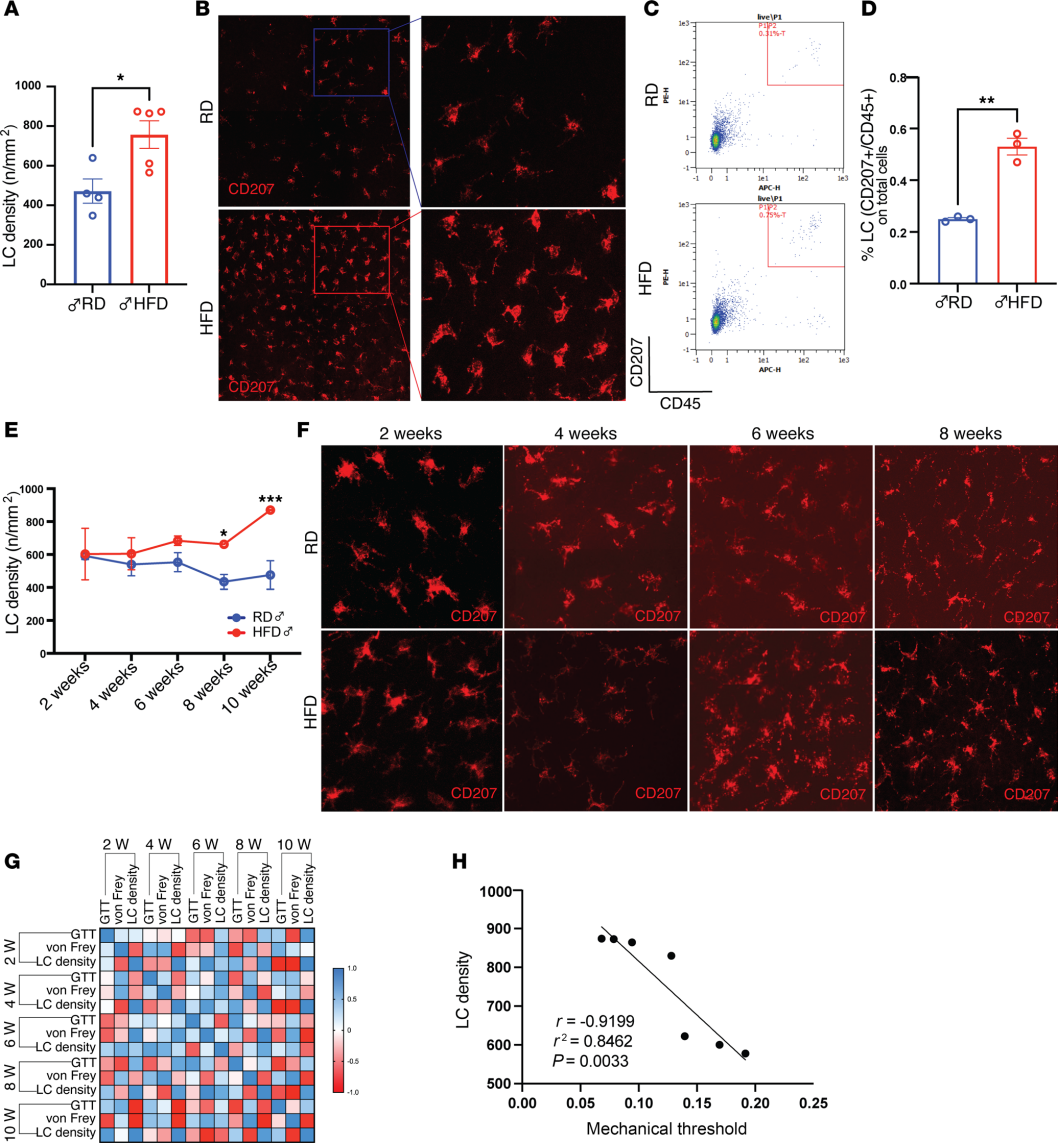
Increased LC density in HFD male mice. (**A** and **B**) Quantification of LC density (number of LCs/mm^2^) in RD and HFD male mice at 10 weeks (10w) (**A**) and representative images (**B**). *n =* 4 animals for RD and *n =* 5 animals for HFD. *n =* 3 sections per animal were acquired and CD207^+^ cells per 0.04 mm^2^ per section area were counted. Unpaired 2-tailed *t* test with Welch’s correction *P = 0.0176. (**C**) MACSQuant Tyto cell sorting of CD207^+^/CD45^+^ gated cells from single cells paw epidermis suspension. PE-CD207 and APC-CD45 antibodies were used to colabel LCs. (**D**) Graph shows the average percentage from 3 replicates of CD207^+^/CD45^+^-sorted cells in RD and HFD. Unpaired 2-tailed t test **P = 0.0010. *n =* 4 animals per diet condition per each replicate. (**E**) Time-course experiment of LC density at 2, 4, 6, 8, and 10w show the progressive increase of LC in HFD. Phenotypic characterization of HFD mice at 2, 4, 6, 8, and 10w obtained from published data (38). Two-way ANOVA for multiple comparisons. Between RD and HFD: 8w *P = 0.0216. 10w **P = 0.0014. Within HFD: 2w versus 10w *P =* 0.0111; 4w versus 10w *P =* 0.0116; 6w versus 10w *P =* 0.0486; 8w versus 10w *P =* 0.0469. 2w: *n =* 3 animals for both RD and HFD. 4w: *n =* 3 animals for both RD and HFD. 6w: *n =* 3 animals for RD and *n =* 4 for HFD. 8w: *n =* 4 animals for RD and *n =* 3 for HFD. 10w: *n =* 4 animals for RD and *n =* 3 for HFD. (**F**) Representative images of LCs CD207^+^ in epidermal sheets of RD and HFD mice at different time points. (**G**) Correlation matrix of HFD male features at different time points (2, 4, 6, 8, 10w) including Glucose Tolerance Test (GTT), mechanical allodynia (vonFrey) and LC density. Red squares indicate negative correlations. 10w vonFrey-LCs: Pearson *r* = –0.9199, *P =* 0.003. (**H**) Negative correlation between LC density and mechanical threshold measured with vonFrey test in 10w HFD mice. Pearson *r* coefficient –0.9199; *r*^2^ = 0.8462; *P* = 0.0033.

To explore a possible role of LCs in PDN, we analyzed LC density in the well-established, and clinically relevant, HFD model of PDN (37–39). Consistent with the literature (40), 10 weeks of HFD induced obesity, glucose intolerance, pain behaviors such as mechanical allodynia, and remodeling of cutaneous innervation (37–41). In this model, we observed an increase in LC density in the whole-mount epidermis of wild-type male mice fed an HFD for 10 weeks, compared to those on a regular diet (RD) (Figure 1, A and B).

To further validate these results, we utilized flow cytometry, a complementary approach commonly used to isolate and quantify immune cells, including LCs (36, 42–45). Consistent with the histological results, flow cytometry revealed a higher proportion of CD45/CD207–double-positive cells in HFD male mice (Figure 1, C and D). Due to the delicate nature of LCs and their low abundance, we used the gentle MACSQuant Tyto sorter (Miltenyi Biotec) to isolate epidermal cells, pooling 3 mice per diet condition for each replicate. The percentage of CD45^+^/CD207^+^ cells in HFD was approximately double that of the RD control (Figure 1D).

To determine whether the observed increase in LCs was due to a localized or systemic change resulting from their activation and proliferation, we labeled LCs in whole-mount ears from both RD and HFD mice. Our analysis showed no significant differences in LC density within the ear epidermis (Supplemental Figure 1A; supplemental material available online with this article; https://doi.org/10.1172/JCI192328DS1). This suggests that, as in patients with PDN where the distal lower extremities are primarily affected, the HFD induces a localized increase in LCs in the paw epidermis.

To evaluate the temporal dynamics of LC changes in response to HFD and their contribution to the development of mechanical allodynia, we measured the LC density at different time points after starting either RD or HFD (2 weeks, 4 weeks, 6 weeks, 8 weeks, and 10 weeks). We observed that the LC density gradually increased over time, becoming significantly higher by week 8 (Figure 1, E and F). We have already shown that, at this time, mice on HFD display mechanical allodynia and small fiber degeneration (38). Correlation analyses revealed that, after 10 weeks on HFD, a higher LC density correlated with a lower mechanical threshold, suggesting that an increased number of LCs corresponded to heightened mechanical pain (Figure 1, G and H). We observed a correlation between the number of LCs and the mechanical threshold in male mice fed a RD; although this was within the normal range of mechanical thresholds (Supplemental Figure 1, B and C).

Because sex differences are fundamental biological variables that influence immune cells, particularly microglia (46–48) and, consequently, immune responses (49) and neuropathic pain (18, 50), we decided to investigate the role of LCs in female mice. We first characterized the phenotype in female mice after 10 weeks on the high-fat diet. Like male mice, female mice on HFD developed obesity, glucose intolerance, and mechanical allodynia (Supplemental Figure 1, D–F). However, histology analysis of paw epidermis uncovered no differences in LC density between RD and HFD female mice (Supplemental Figure 1G). Moreover, RD female mice displayed a LC density higher than that of RD male mice. This suggests that female mice have a higher LC density in the epidermis than male mice (Supplemental Figure 1H).

### LC morphologic remodeling tracks disease progression in patients with painful diabetic neuropathy.

**Figure 2:**
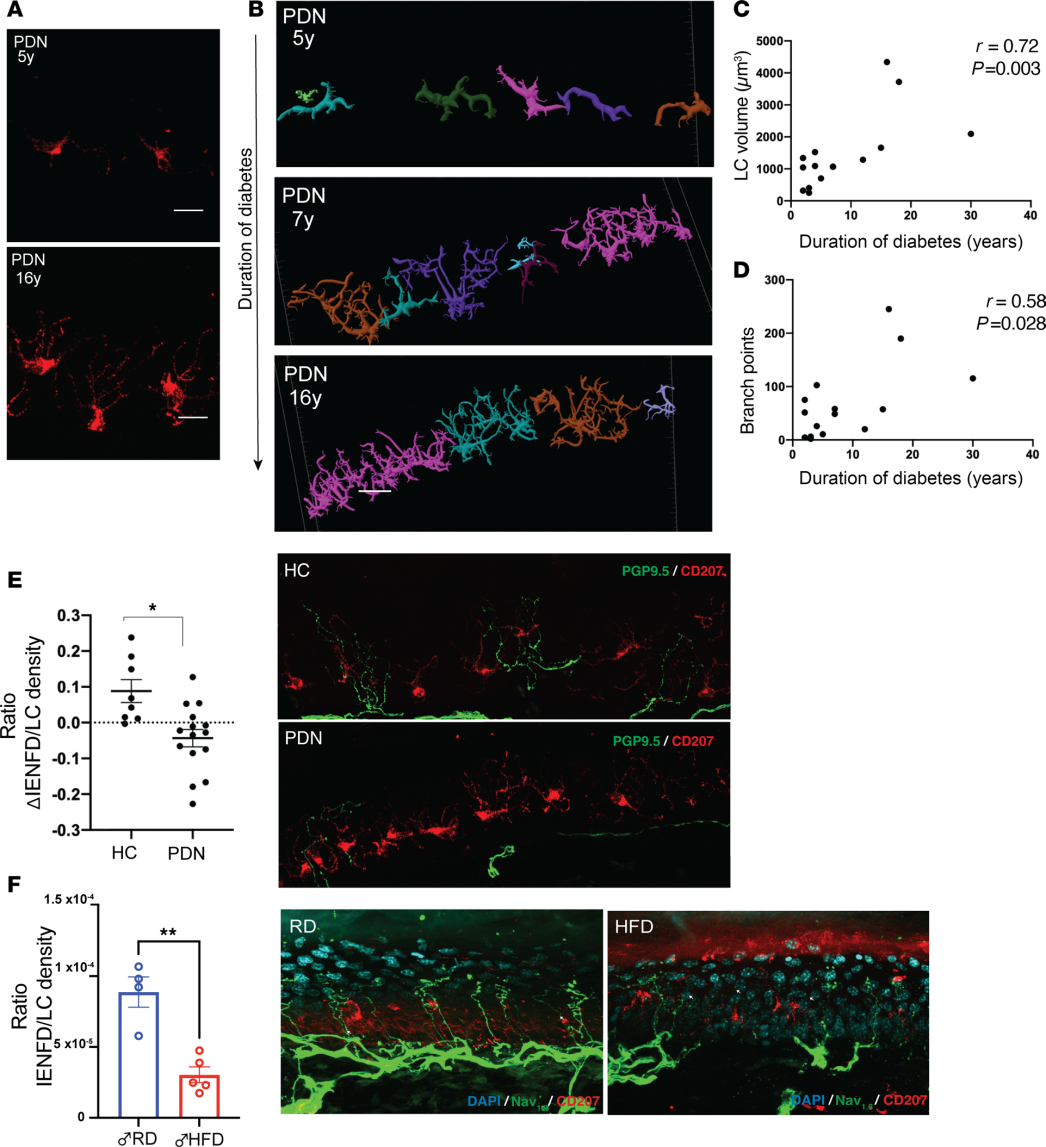
LC morphological analysis in patients with PDN. (**A**) representative images of LCs of patients suffering for diabetes for 5 (left panel) and 16 years (right panel). Scale bar: 15 μm. (**B**) 3D reconstruction of LCs. (**C**) Scatter correlation plots between LCs volume and duration of diabetes. Spearmann correlation *r* = 0.72, *P =* 0.003. (**D**) Scatter correlation plots of number of branch points and duration of diabetes. Spearmann correlation *r* = 0.58, *P =* 0.028. (**E**) ratio between the denervation (IENFD), calculated as deviation of the participants IENFD from the normative value corrected for age and sex, and LC density in healthy participants or patients with PDN. Mann-Whitney U test. *P < 0.05. Middle lines of the plots represent the mean value, while whiskers are SEM. Right panels show representative confocal micrographs of epidermal nerve fibers PGP 9.5^+^ (green) and LCs CD207^+^ (red) in skin sections of healthy participants and patients with PDN. Scale bar: 50 mm. (**F**) Ratio between intraepidermal nerve fibers density and LC density is significantly decreased in HFD male mice. Unpaired 2-tailed *t* test with Welch’s correction **P = 0.0066. Right panels. Representative confocal images (low-magnification) of nociceptive fibers Nav1.8-positive (green) and LCs CD207^+^ (red) in skin sections of RD and HFD male mice.

To enhance the clinical relevance of our findings, we analyzed skin biopsies obtained from clinically well characterized male and female patients with PDN and people who were healthy controls. This included samples from 15 patients with PDN and 9 people who were healthy controls (Table 1). In these human skin biopsies, fixed samples, it was not possible to isolate the epidermis, so we performed 3D reconstructions of LCs in PDN skin samples. Our analysis revealed that both the volume of LCs and the complexity of their arborization processes positively correlated with the duration of diabetes in patients with PDN (Figure 2, A–D). Specifically, our findings indicated that, as diabetes progressed, LCs became larger and their branching structures more complex (Figure 2, C and D). As with other immune cells, which exhibit remarkable plasticity, LC morphology is closely related to their function (51), suggesting that structural and functional changes may reflect more severe phenotypes in patients. Consistent with previous studies (3, 4, 30, 33, 52–54), we found that patients with PDN exhibited a significant reduction in intraepidermal nerve fiber (IENF) density compared with controls, while LC density remained unchanged (Supplemental Figure 2). However, the ratio between IENFD and LC density was reduced in both patients with PDN and in the HFD mouse model of PDN (Figure 2, E and F).

### Targeted depletion of epidermal LCs blocks mechanical allodynia and spontaneous pain in PDN mice.

**Figure 3:**
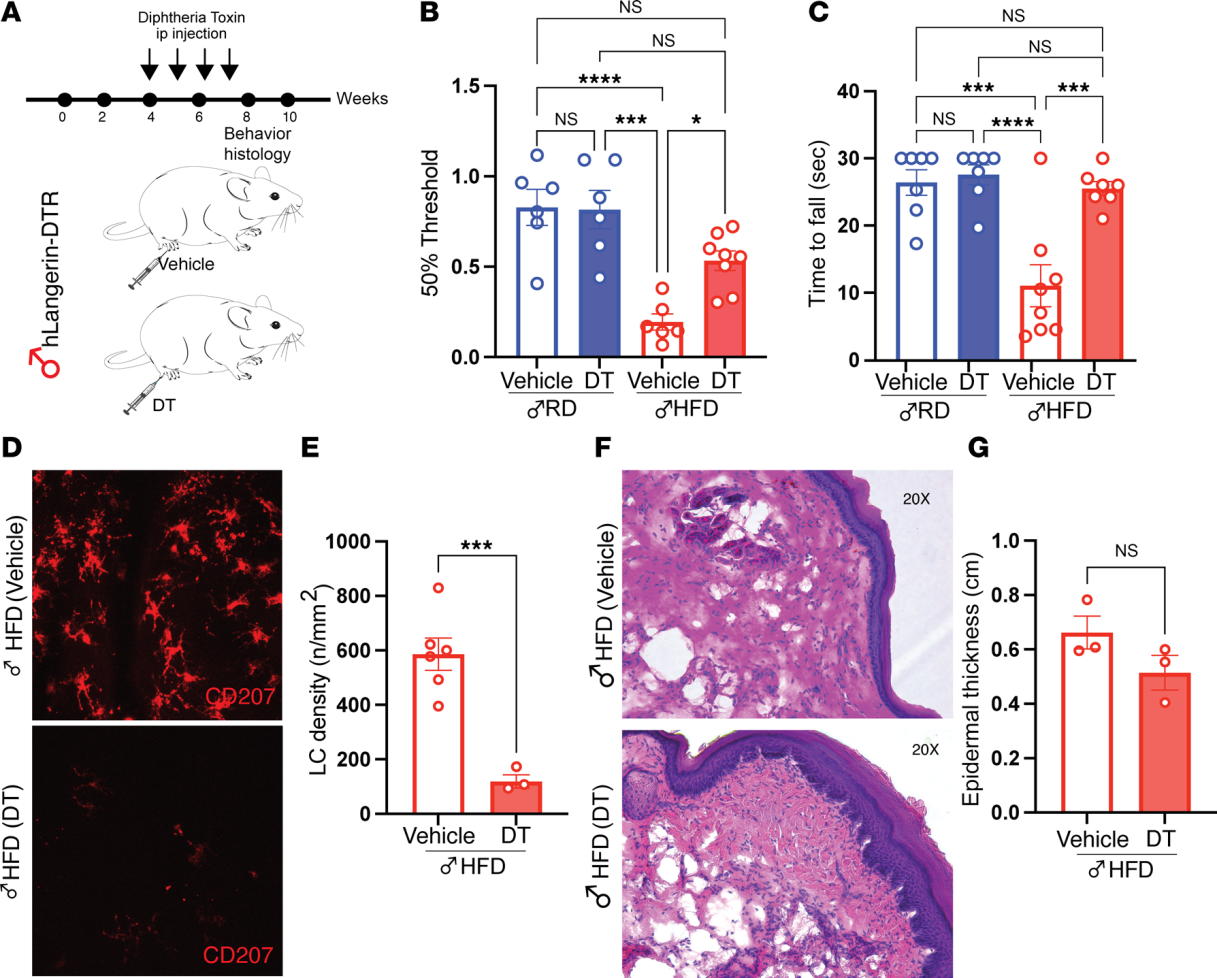
LCs mediate mechanical allodynia and spontaneous pain in HFD. (**A**) Timeline of diphtheria-toxin ablation strategy in hLC-DTR HFD male mice. (**B**) Response to evoked mechanical stimuli threshold. von Frey test shows the recovery of mechanical allodynia in HFD DT-ablated mice. One-way ANOVA for multiple comparison: RD (vehicle) versus HFD (vehicle) ****P < 0.0001; RD (vehicle) versus RD (DT) *P =* 0.9995 (ns); RD (vehicle) versus HFD (DT) *P =* 0.0514 (ns); HFD (vehicle) versus HFD (DT) *P = 0.0213; HFD (vehicle) versus RD (DT) ***P = 0.0001; HFD (DT) versus RD (DT) *P =* 0.0658 (ns). RD (vehicle) *n =* 6; RD (DT) *n =* 6; HFD (vehicle): *n =* 6 HFD (DT): *n =* 8. (**C**) Spontaneous pain behavior measured as time to fall (seconds) is fully recovered by DT-ablation in HFD. One-way ANOVA for multiple comparison: RD (vehicle) versus HFD (vehicle) ***P = 0.0001; RD (vehicle) versus RD (DT) *P =* 0.9824 (ns); RD (vehicle) versus HFD (DT) *P =* 0.9907 (ns); HFD (vehicle) versus HFD (DT) ***P = 0.0003; HFD (vehicle) versus RD (DT) ****P < 0.0001; HFD (DT) versus RD (DT) *P =* 0.9088 (ns). RD (vehicle) *n =* 7; RD (DT) *n =* 7; HFD (vehicle): *n =* 8 HFD (DT): *n =* 7. (**D**) Representative confocal images of paw epidermal sheets of HFD male mice injected with vehicle (0.9% NaCl) or DT 4n g/gr body weight. (**E**) LC density measured as number of CD207^+^ cells/area. Unpaired 2-tailed *t* test with Welch’s correction ***P = 0.0003. HFD (vehicle): *n =* 4. HFD (DT): *n =* 3. (**F**) Representative images of H&E (low-magnification) labeled skin sections of HFD male mice vehicle- and DT- injected. (**G**) quantification of epidermal thickness in HFD mice vehicle- and DT- injected. Unpaired 2-tailed *t* test with Welch’s correction *P =* 0.1681 (ns).

To functionally test in vivo the requirement of LCs for PDN development, we ablated LCs using a transgenic mouse line expressing the diphtheria toxin receptor (DTR) under the control of the langerin/*Cd207* promoter (36, 55). We administered diphtheria toxin (DT) observing that weekly injections, from week 4 to week 8, effectively depleted LCs in DT-treated mice (Figure 3A). Indeed, histological analysis of paw epidermal sheets confirmed a 70% reduction in LC density 2 days after the final DT injection compared with control mice injected with vehicle (0.9% NaCl) (Figure 3, D and E).

Behavioral testing revealed that repeated DT injections prevented the development of mechanical allodynia in the HFD male mice (Figure 3B). In contrast, HFD mice that received vehicle treatment maintained robust allodynia (Figure 3B), indicating that LCs are necessary for the development of mechanical allodynia in the HFD model of PDN. On the contrary, DT-induced LC ablation did not affect the response to mechanical stimulation in RD mice (Figure 3B). Additionally, assessment of spontaneous pain through a cage-lid hanging test (56) showed that HFD male mice developed spontaneous pain, which was reversed by LC ablation (Figure 3C). Histological analysis confirmed that the DT-mediated strategy effectively removed LCs (Figure 3, D and E) without altering the histological organization of the epidermis (Figure 3, F and G). Quantification of IENF density of PGP9.5-positive fibers revealed that LC depletion could potentially influence cutaneous fiber innervation in HFD male mice, although no statistically significant differences were detected (Supplemental Figure 3A). To assess whether LC loss affected a defined subset of DRG sensory neurons previously linked to these cells (36), we generated a double-transgenic mouse line by crossing *Mrgprd*-eGFP mouse line (57) with hLC-DTR mice (*Mrgprd*eGFP-hLCDTR). As observed in hLC-DTR mice, DT-mediated LC depletion in *Mrgprd*eGFP-hLCDTR mice prevented the development of HFD-induced mechanical allodynia (Supplemental Figure 3, B and C), without affecting dermal cell populations (Supplemental Figure 3D). Quantification of IENF density of *Mrgprd*-eGFP-positive fibers revealed no significant differences between DT- and vehicle-treated mice (Supplemental Figure 3E), highlighting the difficulty of capturing dynamic processes as fiber degeneration and regeneration at this PDN stage.

We also evaluated the impact of LC depletion in female HFD mice. We found that, as opposed to males, in female mice, the loss of LCs induced by DT did not prevent mechanical allodynia (Supplemental Figure 3, F and G), suggesting that sex-dependent mechanisms influence the role of LCs in PDN. Together, these results demonstrated that epidermal LCs are essential for maintaining mechanical allodynia and spontaneous pain in the male HFD model of PDN.

### Single-cell transcriptional profiling reveals sexually dimorphic signatures in mouse epidermis.

**Figure 4:**
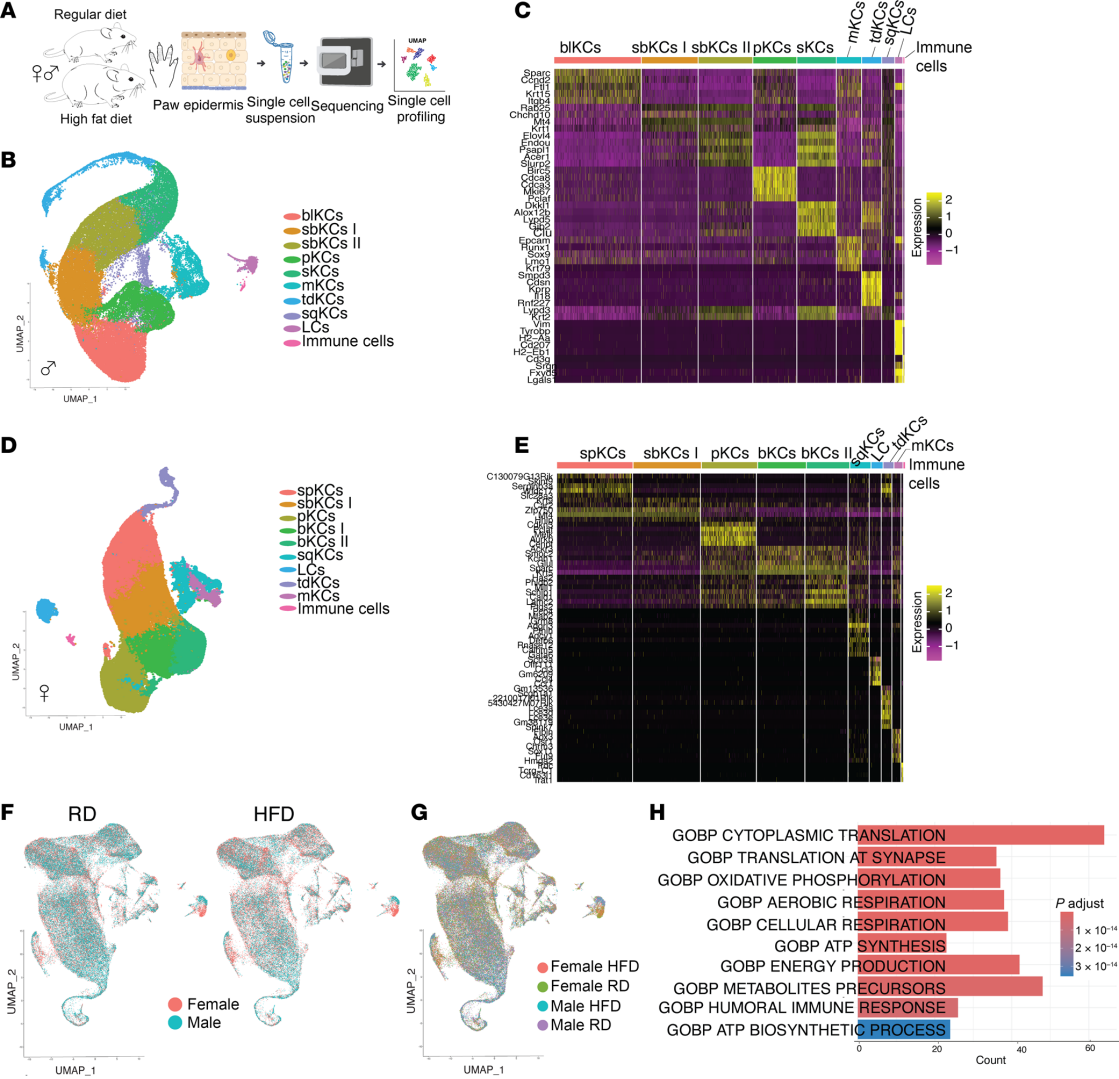
scRNA-seq of paw epidermis of RD and HFD male and female mice. (**A**) Schematic workflow of scRNA-seq of paw epidermis of RD and HFD male and female mice. (**B**) UMAP plot visualization of all 10 clusters identified in scRNA-seq data of RD and HFD male paw epidermis. (**C**) Heatmap of top 5 differentially expressed genes in each cluster. (**D**) UMAP dimensionality reduction of RD and HFD scRNA-seq paw epidermis of female mice shows 10 distinct clusters identified by the expression of differentially expressed genes (DEGs). (**E**) Heatmap of top 5 DEGs expressed in each cluster. (**F** and **G**) UMAP dimensionality reduction of integration analysis of RD and HFD both male and female scRNA-seq datasets using anchor-based CCA integration. (**H**) Enrichment pathways analysis using EnrichR comparing HFD LCs in male and female datasets.

**Figure 5:**
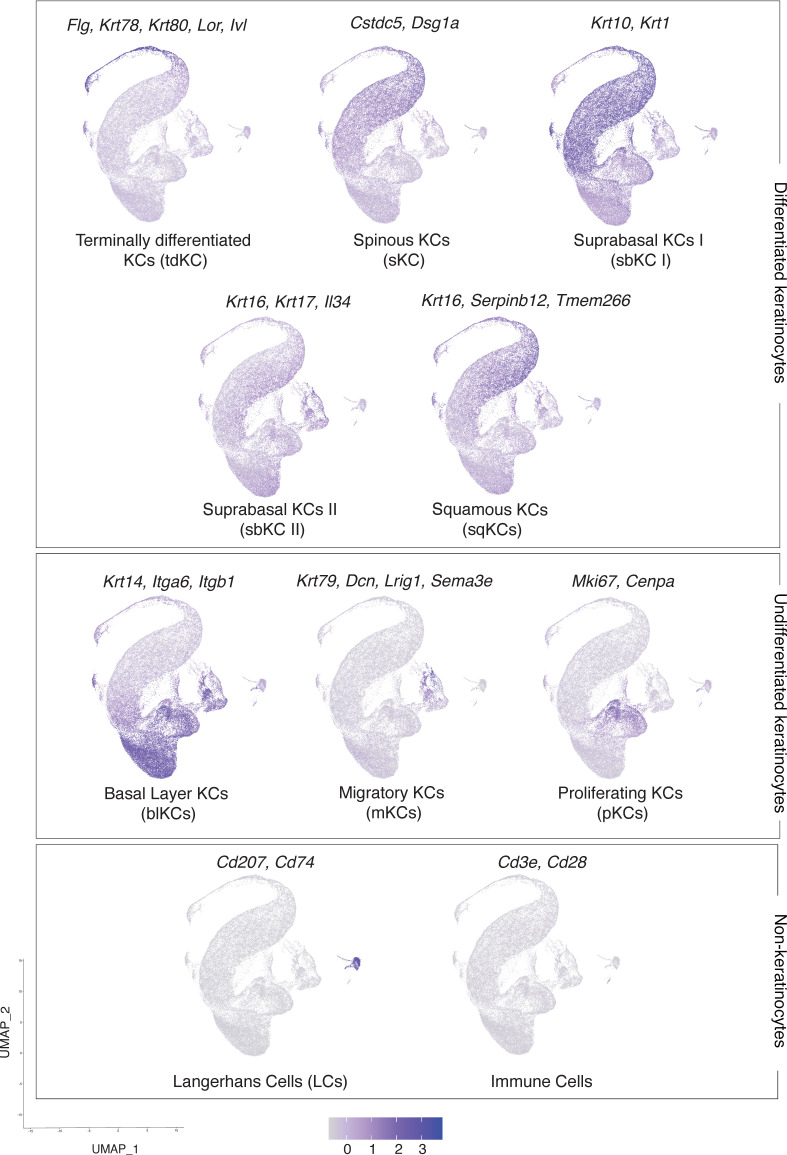
Profiling of epidermal cell clusters. Feature plot of the most representative gene markers used to identify each epidermal cluster. Terminally differentiated Keratinocytes (tdKCs) identified by *Flg*, *Krt78, Krt80, Lor,* and *Ivl*. Spinous Keratinocytes (sKCs) identified by *Cstdc5* and *Dsg1a*. Suprabasl keratinocytes type I (sbKCs I) identified by *Krt10* and *Krt1*. Suprabasal Keratinocytes type II (sbKCs II) identified by *Krt16, Krt17,* and *Il34*. Squamous Keratinocytes (sqKCs) identified by *Krt16*, *Serpinb12,* and *Tmem266*. Basal Layer Keratinocytes (blKCs) identified by *Krt14, Itga6,* and *Itgb1*. Migratory Keratinocytes (mKCs) identified by *Krt79, Dcn, Lrig1* and *Sema3e*. Proliferating Keratinocytes (pKCs) identified by *Mki67* and *Cenpe*. Nonkeratinocyte cells LCs identified by *Cd207* and *Cd74*; Immune Cells identified by *Cd3e* and *Cd28*.

To investigate the molecular mechanisms underlying LC-dependent neuropathic pain in the HFD mouse model of PDN, we performed an unbiased single-cell RNA-seq (scRNA-seq) of the paw epidermis in male mice. Mice were fed either an RD (RD *n* = 3) or an HFD (HFD *n* = 3) for a duration of 10 weeks (Figure 4A). After separating the epidermis from the dermis, we obtained a high-viability single cell–suspension from each sample, collecting 40,500 cells from the RD epidermis and 28,950 cells from the HFD epidermis. Single cells were sequenced using the 10x Genomics Chromium platform. To remove doublets and low-quality cells, we filtered out cells with fewer than 200 or more than 6,000 features and those containing more than 5% mitochondrial reads (Supplemental Figure 4, A–G). We identified 10 distinct clusters based on the shared nearest neighbor (SNN) clustering algorithm in Seurat. We then visualized the different clusters using the 2-dimensional Uniform Manifold Approximation and Projection (UMAP) method (Figure 4B). Based on known marker expression and the top 5 differentially expressed genes (DEGs) for each cluster (Figure 4C), we identified 8 keratinocyte subpopulations representing different stages of differentiation and 2 groups of nonkeratinocytes. As keratinocytes are the most abundant cell type in the epidermis, we used multiple markers to define their differentiation stages (Figure 5 and Supplemental Figure 5A). Feature plots illustrated the distribution of keratinocyte markers, highlighting terminally differentiated keratinocytes, usually located in the upper layer of the epidermis corresponding to the stratum corneum, differentiated keratinocytes including spinous, suprabasal, and squamous keratinocytes, undifferentiated basal epithelium keratinocytes mainly expressing *Keratin14* (*Krt14*), migratory keratinocytes and keratinocytes with higher expression of *Mki67*,a distinctive marker of proliferation (Figure 5 and Supplemental Figure 5, A and B). Specifically, we identified: (a) terminally differentiated keratinocytes (tdKCs) expressing *Flg, Krt78, Krt80, Lor* and *Ivl*; (b) spinous keratinocytes (sKCs) expressing *Cstdc5* and *Dsg1a*; (c) suprabasl keratinocytes type I (sbKCs I) and (d) type II (sbKCs II) specified by *Krt10* and *Krt1*, and *Krt16*, *Krt17* and *Il34*, respectively; (e) squamous keratinocytes (sqKCs) expressing *Krt16, Serpinb12* and *Tmem266*. To define undifferentiated KCs, we used *Krt14, Itga6* and *Itgb1* for (f) basal layer keratinocytes (blKCs); *Krt79, Dcn, Lrig1* and *Sema3e* for (g) migratory Keratinocytes (mKCs) and, lastly, *Mki67* and *Cenpa* for (h) proliferating keratinocytes (pKCs) (Figure 5). Furthermore, we used the high and selective expression of *Cd207, Cd74, Cd28,* and *Cd3e* to identify the remaining no-keratinocytes as LCs (i) and immune cells (j), respectively (Figure 5).

Next, we extended the single-cell transcriptomic analysis to the epidermis of female mice in the HFD model of PDN. We performed scRNA-seq of the paw epidermis of female RD (RD *n =* 3) and HFD (HFD *n =* 3) mice (Figure 4D). A high-viability single cell–suspension containing 33,175 cells from RD epidermis and 31,047 cells from HFD epidermis, was analyzed for quality control and filtration steps to improve data reliability and exclude low-quality or dying cells (Supplemental Figure 4, H and I). Like male scRNA-seq data, 10 clusters were identified in female mice, primarily keratinocytes, immune cells, and LCs (Figure 4, D and E). To assess sex-dependent molecular differences, we conducted a comprehensive comparison of male and female scRNA-seq datasets under both RD and HFD conditions. We integrated both scRNA-seq datasets while correcting for batch effects across sexes and diet conditions, thereby improving comparability between samples (Figure 4F). The integrated UMAP analysis revealed extensive overlap between male and female datasets, indicating a strong correspondence and comparable representation of the distinct clusters across sexes (Figure 4G and Supplemental Figure 4J). Focusing on the LC cluster, we performed a web-based gene set analysis (58, 59) to identify diet-enriched pathways in both sexes. We found that the LC cluster showed upregulation in pathways linked to protein translation and synaptic signaling only in male HFD mice (Figure 4H).

Finally, we validated selected cluster markers in paw skin sections of RD and HFD mice. KRT10 was used as a marker of epithelial differentiation, while KRT14 labeled basal cells in contact with the basement membrane (Supplemental Figure 4K). Epidermal LCs were labeled using CD207 in paw skin sections from RD and HFD male mice (Supplemental Figure 4L).

### Transcriptional profiling reveals inflammatory and axonal guidance programs in LCs from PDN male mice.

**Figure 6:**
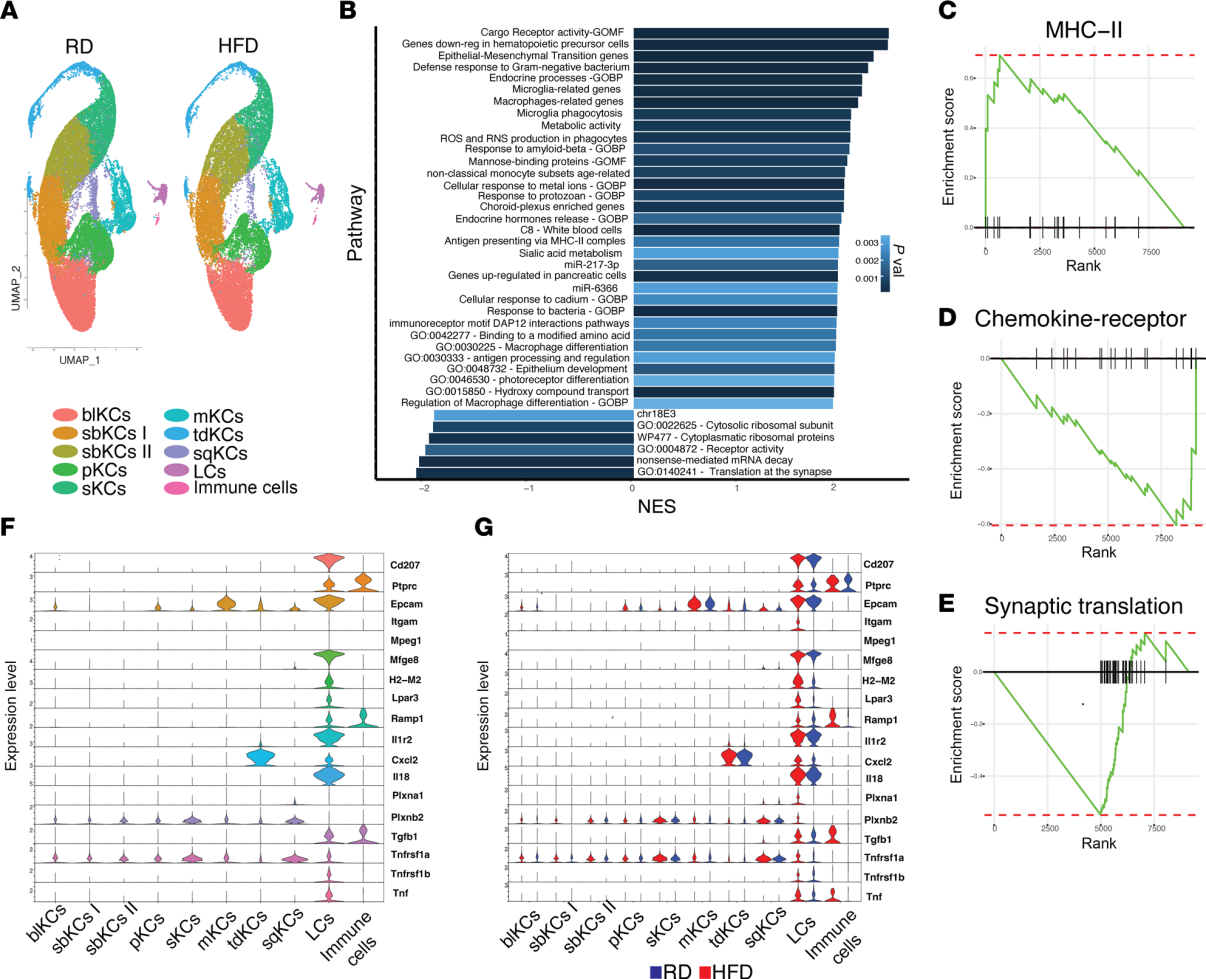
GSEA of LC cluster. (**A**) UMAP plots of the scRNA-seq of the 2 diet conditions, RD and HFD. (**B**) Plot shows GSEA of Langerhans cells cluster identifies enriched sets of genes with positive and negative NES value (|NES| > 1.88). (**C**–**E**) examples of major enriched pathways with positive NES showing the enrichment in MHC-II–related genes (**C**) and negative NES (**D**–**E**. Statistics were derived using the fgsea package in R. Adjusted *P* value results from Benjamini-Hochberg correction. (**F** and **G**) Stacked violin plot showing the expression of genes implicated in axonal guidance, such as *Plxna1* and *Plxnb2*, and inflammatory response. Combined (**F**) and split (**G**) by diet conditions gene expression (RD, blue; HFD, red).

To define the transcriptional programs altered across epidermal cell types in the HFD model of PDN, we performed a comparative clustering analysis of scRNA-seq datasets from RD- and HFD-fed mice. This approach revealed shifts in molecular features across several epidermal populations in male mice (Figure 6A). Although the integrated male-female analysis showed comparable overall epidermal cellular composition, LCs emerged as the most sex-divergent cell type under HFD conditions (Figure 4, F and G). Male LCs, but not female LCs, exhibited robust transcriptional remodeling in response to diet. We therefore focused subsequent analyses on defining the pathways specifically altered in LCs from PDN male mice fed a HFD compared with RD-fed controls.

Gene set enrichment analysis (GSEA) (60) using the Mouse MSigDB (61) on Seurat-derived DEGs (see methods) identified discrete functional programs enriched in male HFD LCs. Pathways with positive normalized enrichment scores (NES) were dominated by MHC class II–mediated antigen-presentation signatures, whereas pathways with negative NES values mapped to synaptic protein networks and inflammatory signaling modules (Figure 6, B–E, and Supplemental Figure 6, A and B).

Integration of these transcriptional signatures with prior literature (62) further highlighted coordinated upregulation of immune-response genes and axonal-guidance pathways in male HFD LCs. Notably, the axon-guidance receptors *Plxnb2* and *Plxna1* were significantly increased, along with chemokine-related and other immune signaling genes (Figure 6, F and G). These findings indicate that HFD exposure in PDN males elicits a combined antigen-presenting, inflammatory, and neuroimmune remodeling program in LCs.

In contrast, the LC cluster from female HFD mice exhibited a nonoverlapping DEG profile characterized by increased expression of the sodium channel *Scn3a* and the purinergic receptor *P2rx2* (Supplemental Figure 6C). Comparative analysis of male and female HFD datasets confirmed that LC transcriptional responses to diet are highly sex specific and involve distinct molecular pathways (Supplemental Figure 6E).

**Figure 7:**
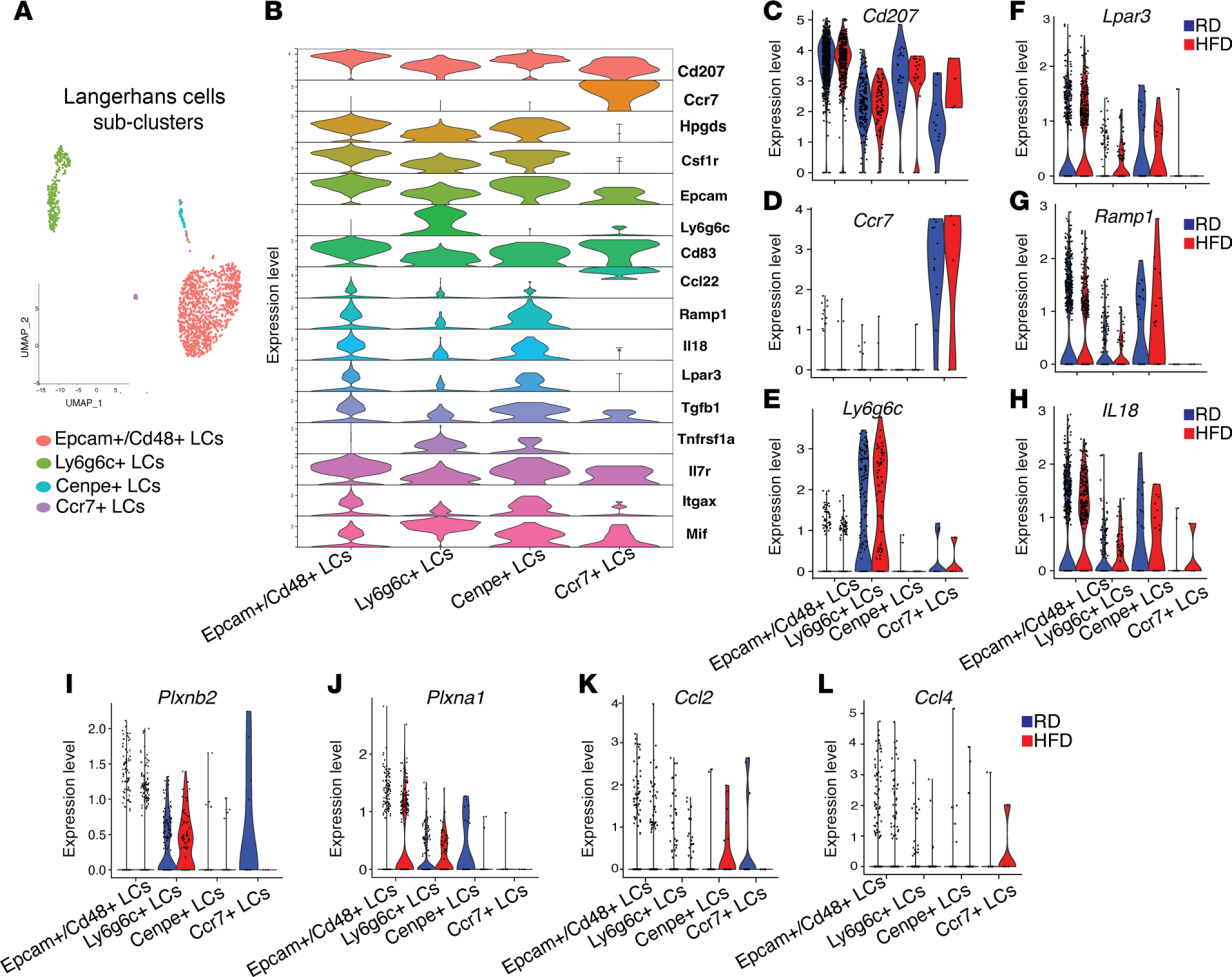
LC subclustering analysis and gene expression profiles. (**A**) UMAP plots of the LC subclustering showing 4 distinct groups. (**B**) Stacked violin plot of marker genes adopted to identify LC subclusters. (**C**–**H**) ViolinPlot showing the expression of *Cd207* (**C**), *Ccr7* (**D**), *Ly6g6c* **E**), *Lpar3* (**F**), *Ramp1* (**G**) and *IL18* (**H**) in LCs subclusters. (**I**–**L**) ViolinPlots show the expression and distribution of plexins molecules *Plxnb2* (**I**) and *Plxna1* (**J**) and chemokine molecules (**K**) *Mcp1/Ccl2* and (**L**) *Mip-1b/Ccl4* in LC subclusters.

Given the known cellular and functional diversity of LCs (45, 63–65), we conducted a subcluster analysis of the major cluster of these cells. This analysis revealed 4 transcriptionally distinct subtypes (Figure 7A), each characterized by unique markers (Figure 7B and Supplemental Figure 7, A and B). Each of these LC subtypes may carry out different functions in PDN. We identified (a) *Epcam*^+^*/Cd48*^+^ LCs, (b) *Ly6g6c^+^* LCs, (c) *Cenpe*^+^ LCs, and (d) *Ccr7*^+^ LCs. All 4 clusters expressed the common LC marker *Cd207* (Figure 7, A–C). Specifically, *Epcam*^+^*/Cd48*^+^ LCs were identified by the expression of *Cd48*, which was elevated in HFD (Supplemental Figure 7, A and B). *Cd48*, a member of the signalling lymphocyte activation molecule family, is usually associated with heightened activation of immune cells (66), including LCs (44). *Ly6g6c*^+^ LCs indicated a monocyte-derived origin (Figure 7E), and, similar to resident macrophages, *Ly6g6c*^+^ LCs might be recruited from the blood, contributing to the higher number of cells observed in the HFD epidermis (63, 67). *Cenpe*^+^ LCs (Supplemental Figure 7, A and B) suggested that a small number of LCs might undergo local cell division and proliferation (68). *Ccr7*^+^ LCs represented a migratory phenotype (42, 69) (Figure 7D). We also detected the transcripts of *Tnfa*, *Tgfb1,* *Ccl22* (Supplemental Figure 7, A and B), and *Lpar3*, markers of nonpeptidergic nociceptors type 1 (NP1) (70, 71), which were upregulated in HFD (Figure 7F), as well as *Ramp1*, coreceptor of calcitonin gene-related peptide receptor (CGRP) (72) (Figure 7G). HFD LCs subtypes also expressed high levels of axonal guidance molecules such as *Plxnb2* and *Plxna1* (Figure 7, I and J). Moreover, we found that inflammatory mediators, specifically IL-18 (Figure 7H) and the chemokine *Ccl2*, were upregulated in HFD LCs (Figure 7, K and L). Notably, these inflammatory mediators, especially CCL2, have been implicated in mediating neuropathic pain (73, 74).

In female LCs, we identified 4 subtypes: (a) *Epcam*^+^*/Cd48*^+^ LCs, (b) *Ly6g6c* ^+^ LCs, (c) *Ccr7*^+^ LCs, and (d) *Cenpe*^+^ LCs, which are similar to the subtypes found in males (Supplemental Figure 7, C and D). However, axonal guidance molecules such as Plxnb2 and Plxna1, as well as the chemokine CCL2, were not detected (Supplemental Figure 7E). This indicates that female LCs may interact with sensory fibers through different mechanisms.

### Ligand-receptor analysis reveals neuro-immune crosstalk via Semaphorin-plexin and chemokine pathways.

**Figure 8:**
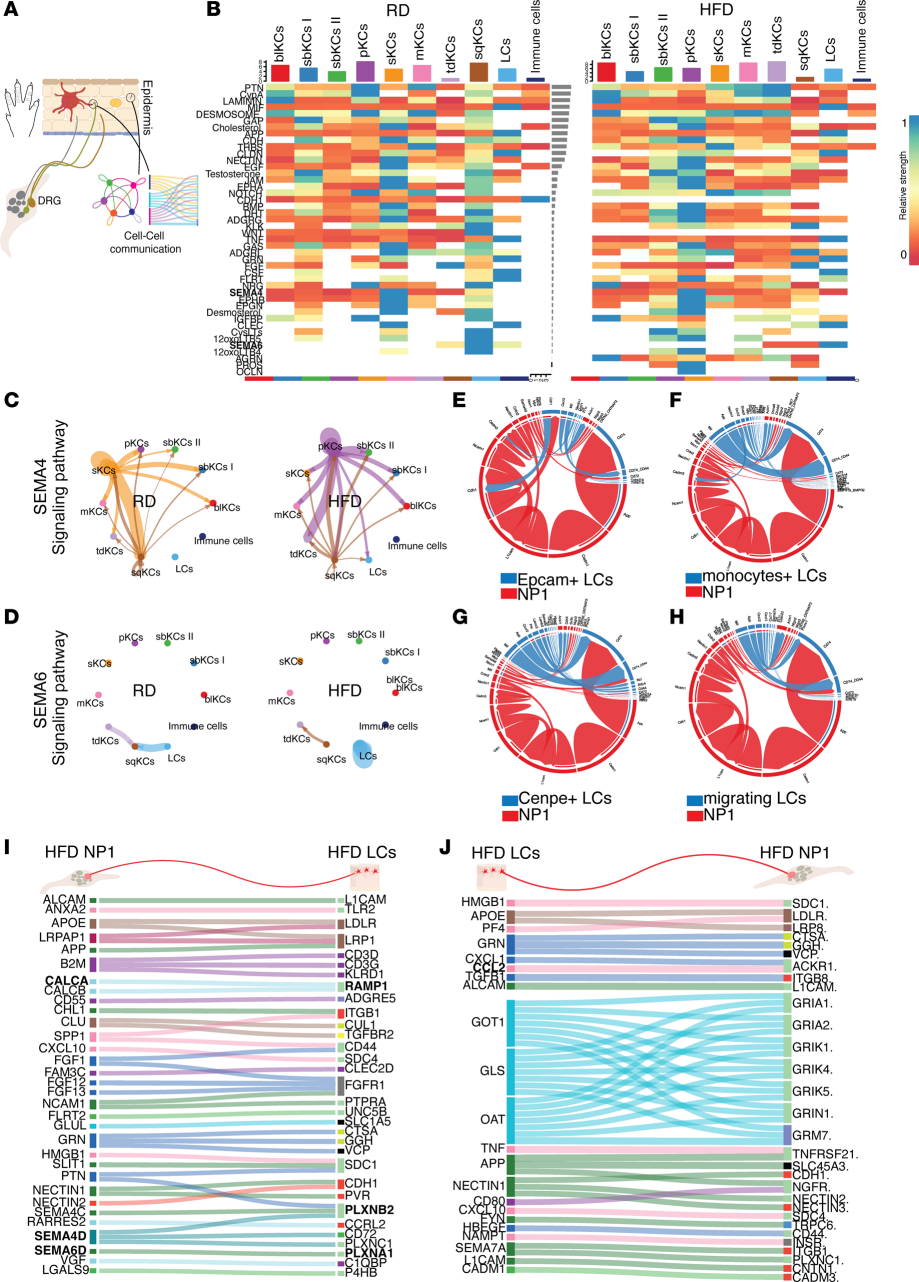
Cell-cell communication between LCs and DRG sensory neurons. (**A**) Schematic cartoon of cell-cell ligand-receptor network analysis performed using CellChat and Interactome analysis platforms (**B**) Comparison of cell-cell communication between RD and HFD using Heatmap plot. Highlighted in bold *Sema4* and *Sema6* signaling pathways (**C** and **D**) Circle plots show *Sema4* and *Sema6* signaling pathways in RD and HFD. (**E**–**H**) Chord grams of integrated analysis of HFD LC subclusters and HFD NP1 DRG. (**I** and **J**) interactome analysis platform to explore the communication between HFD LC cluster and HFD NP1 DRG.

To investigate how LCs communicate with surrounding cells and nerve afferents in the epidermis, we applied 2 complementary computational platform analyses to our scRNA-seq datasets. We used CellChat (75) and the interactome analysis platform (76) to infer ligand-receptor–mediated signaling (Figure 8A). Focusing on the male dataset, CellChat indicated that both the number of inferred cell-cell communications and the strength of these interactions were increased in HFD mice compared with RD controls (Supplemental Figure 8, A and B). Pathway analysis showed dramatic differences between RD and HFD conditions, including among keratinocyte clusters with changes in the pleiotrophin (*Ptn*) or Epidermal Growth Factor (*Egf*) pathways, which are known to be upregulated in wound repair and healing (77), or in cell adhesion molecules (*Nectin*) (Figure 8B). Moreover, among pathways involved in neuronal communication, semaphorin signaling, especially *Sema6* and *Sema4* families, was differentially regulated in HFD LCs (Figure 8, C and D). Integrating epidermal scRNA-seq data with DRG (57) datasets from RD and HFD revealed upregulation of Plxnb2 and Plxna1 receptors in HFD LCs, while their ligands, Sema4d and Sema6d, respectively, were expressed by HFD nonpeptidergic nociceptor type 1 (NP1) DRG neurons (Supplemental Figure 8, C–F). Further analysis of LC subclusters and NP1 DRG cells expressing *Mrgprd* indicated that communication between LCs and NP1 also relies on synaptic contact molecules, such as neurexins (*Nrxn*) (Figure 8, E–H, and Supplemental Figure 8, G–J), suggesting that LCs may establish synaptic-like contacts with sensory afferents in the epidermis. Using the interactome analysis platform (76) with LCs as the receptor component, we confirmed that these cells communicate with NP1 DRG through Sema4- and Sema6-signaling pathways (Figure 8I). In-situ RNA scope in DRG sections also confirmed the expression of *Sema4d* and *Sema6d* in NP1 *Mrgprd*^+^ DRG neurons (Supplemental Figure 8, K and L), supporting putative LC-NP1 communication through these pathways. Interestingly, when the ligand-receptor expression was inverted, LCs were predicted to interact with NP1 neurons via the chemokine CCL2 pathway (Figure 8J).

### Langerhans cells modulate nociceptive responses via CCL2-CCR2 chemokine signaling.

**Figure 9:**
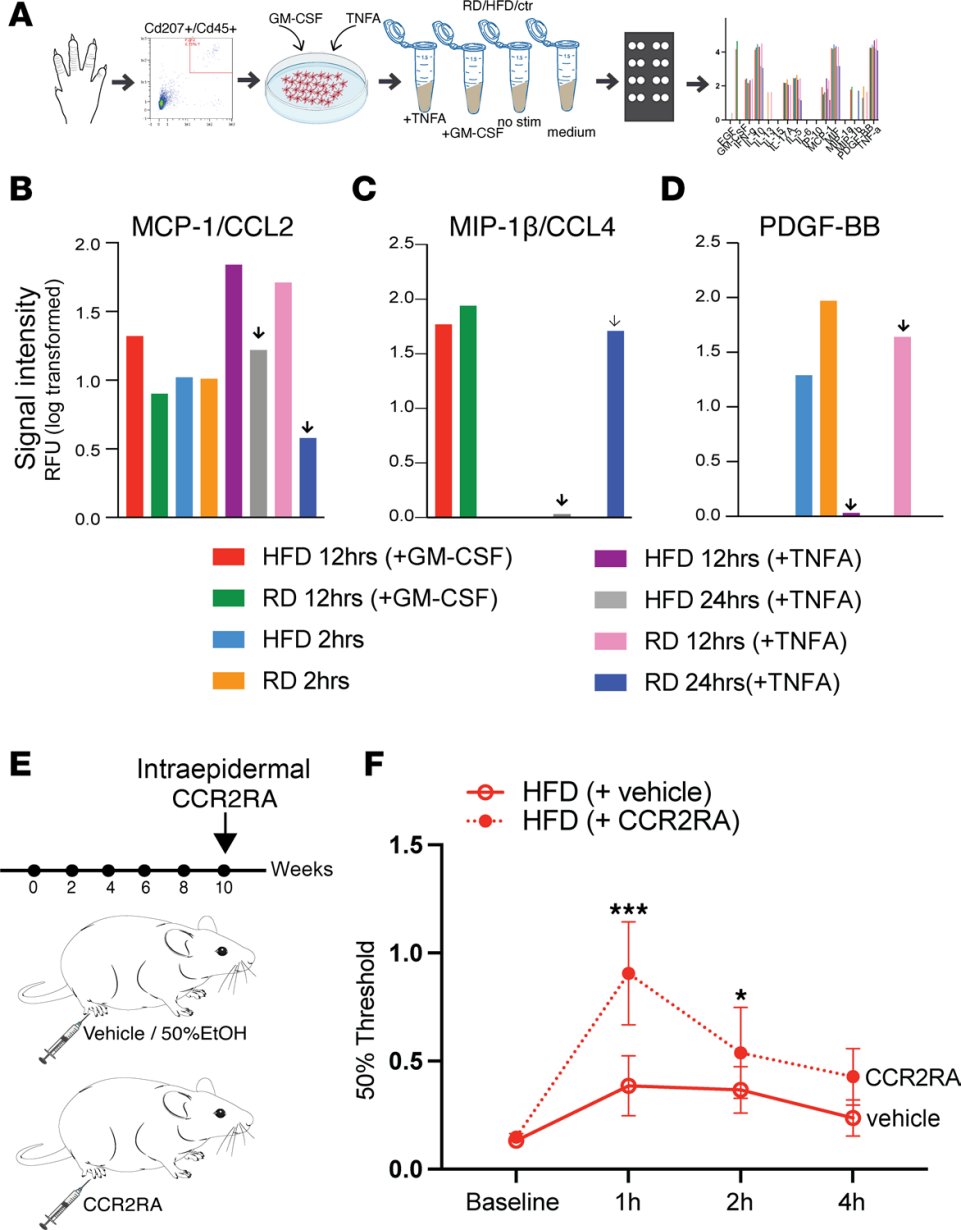
High-multiplexing cytokine profile of LCs in RD and HFD. (**A**) Representative workflow of cytokine profile assay (**B**–**D**) Barplots show inflammatory molecules detected in the multiplexed proteomic profiling. Black arrows indicate differences for diet and treatment condition. (**E** and **F**) Intraplantar injection of CCR2RA improves mechanical allodynia in HFD male mice. Mixed effects analysis for multiple comparisons HFD +CCR2RA: baseline versus 1h ***P = 0.0001, baseline versus 2h *P = 0.0176 HFD vehicle *n =* 6, HFD CCR2RA *n =* 8.

As key players in the innate immune response (26), LCs respond to activation by releasing proinflammatory cytokines and nitric oxide, factors that can sensitize epidermal DRG axonal afferents (18). To define how LC secretory programs are altered in PDN, we performed multiplexed proteomic profiling on sorted CD45^+^/CD207^+^ LCs from RD and HFD male mice (Figure 9A). Consistent with their immunologic function, LCs secreted TNFA, GMCSF, IL-10, and MIF (Supplemental Figure 9, A and B). Strikingly, monocyte chemoattractant protein-1 (MCP-1/CCL2) remained elevated in HFD LCs following TNFA stimulation for 24 hours (Figure 9B), confirming at the protein level that chemokine CCL2 is upregulated in HFD-associated LC dysfunction. In contrast, macrophage inflammatory protein-1β (MIP-1β/CCL4) was selectively secreted by RD LCs under the same inflammatory conditions (Figure 9C). We also identified dysregulation of platelet-derived growth factor-BB (PDGF-BB), a potent mitogenic signal, in HFD LCs (Figure 9D), highlighting broader disruption of LC secretome pathways in PDN.

Given the well-defined role of CCL2-CCR2 signaling in neuropathic pain (73, 74), we next evaluated its causal contribution to PDN. Local intraplantar administration of a selective CCR2R antagonist (CCR2RA) transiently but robustly reversed mechanical allodynia in HFD mice for approximately 2 hours (Figure 9, E and F), without affecting LC density (Supplemental Figure 9C). Together, these findings demonstrate that peripheral CCL2-CCR2 signaling is required for the development of mechanical allodynia in the HFD model of PDN and identify LCs as an essential source of this pronociceptive chemokine.

## Discussion

In this study, we identified a previously unrecognized role for epidermal LCs in PDN and defined a peripheral, chemokine-dependent neuron-immune communication pathway at the skin-nerve interface. Using a clinically relevant HFD mouse model together with analyses of skin biopsies from patients with well-characterized PDN, we showed that epidermal LCs contribute to nociceptor sensitization through sex-dependent immune programs. In male mice, LC density increased in paw epidermis in parallel with the development of mechanical hypersensitivity. In human PDN skin, LCs exhibited progressive morphological remodeling, including increased volume and dendritic complexity, which correlated with disease duration. Conditional LC depletion prevented both evoked mechanical allodynia and spontaneous pain-like behavior in male HFD mice, establishing a causal role for LCs in pain behavior. Single-cell transcriptomic, ligand-receptor, and secretome analyses revealed HFD-induced neuroimmune remodeling of male LCs, including inflammatory and axon-guidance pathways and increased secretion of the chemokine CCL2. Consistent with this mechanism, local pharmacological blockade of CCR2 attenuated mechanical allodynia. Together, these findings demonstrate that epidermal LCs can actively drive peripheral nociceptor sensitization and neuropathic pain in PDN via chemokine-dependent neuroimmune signaling.

LCs constitute approximately 2%–5% of epidermal cells and are classically recognized for their role in immune surveillance, extending dynamic dendritic processes between keratinocytes to sample antigens (25, 51, 78, 79). Beyond this sentinel function, LCs regulate innate immune responses and coordinate adaptive immune cell recruitment through cytokine and chemokine release under both physiological and pathological conditions (21, 26, 62, 80, 81). Although LCs have been implicated in several inflammatory skin disorders (26, 45, 55, 79), their contribution to PDN has not previously been explored. In contrast, the prevailing view of PDN pathogenesis has focused largely on intrinsic metabolic injury to sensory neurons and dorsal root ganglia (82). Our findings expand this framework by providing direct evidence that epidermal LCs play a critical and previously unappreciated role in mediating neuropathic pain in PDN.

To determine whether LC accumulation in the epidermis is functionally relevant to PDN, we transiently ablated LCs using a genetic diphtheria toxin receptor (DTR) strategy while monitoring pain behaviors in HFD-fed mice. Conditional DTR expression under the control of the *Cd207* (langerin) promoter enabled selective LC depletion following repeated low-dose diphtheria toxin (DT) administration (4 ng/g body weight) (36, 55). A key interpretive issue in the field is that some depletion paradigms using supramaximal DT protocols that can themselves elicit pain behavior (34). Consistent with prior reports (36, 55), the low-dose, repeated regimen we used in our study effectively reduced epidermal LC density without inducing mechanical hypersensitivity in regular diet controls. Strikingly, LC depletion selectively prevented HFD-induced mechanical allodynia and spontaneous pain-like behavior in male mice. These data argue that LCs are not simply markers of inflammatory diabetic skin but are necessary contributors to the pain phenotype. Notably, this effect was sex dependent: female mice developed metabolic dysfunction and mechanical allodynia but did not exhibit increased LC density, and CD207^+^ cell depletion did not prevent pain behavior.

To explore molecular mechanisms underlying LC-dependent neuropathic pain, we performed unbiased single-cell RNA-seq of the epidermis in male and female mice. To our knowledge, this represents the first single-cell transcriptomic analysis of epidermal immune cells in an HFD model of PDN. This approach revealed pronounced sex-specific transcriptional responses in LCs following HFD exposure, consistent with prior reports of sexually dimorphic immune contributions to neuropathic pain, particularly those described for microglia (46–48, 83). In male mice, LC transcriptional programs were enriched for inflammatory and chemokine signaling pathways, including CCL2/CCR2, as well as neuroimmune communication pathways. In contrast, female LC transcriptomes showed a distinct response to HFD, lacking the male-associated *Ccl2/Plxn* program and instead upregulating alternative genes, including ion channel transcripts. Integrated analysis of male and female datasets confirmed overlap in overall epidermal cellular composition while identifying LC-selective programs enriched in male HFD mice, including pathways related to protein translation, synaptic signaling, and chemokine biology. These findings suggest that PDN pain can be sustained by mechanistically distinct neuroimmune circuits in males versus females, consistent with broader evidence that immune contributions to pain are sex dependent (18, 46, 50, 84–86). A limitation of this study is that female-specific mechanisms underlying neuropathic pain remain undefined. Future studies will be needed to define peripheral cell types and mediators that compensate for LC-dependent mechanisms in females and to determine whether sex differences reflect divergent LC states, differences in neuron-immune connectivity, or engagement of compensatory pathways.

Among candidate mechanisms, mechanistically, our data converged on CCL2-CCR2 signaling as a major LC-linked pronociceptive pathway in male PDN mice. CCL2/CCR2 signaling has been broadly implicated in neuropathic pain (87–93) and can increase sensory neuron excitability via actions on DRG neuronal somata (74, 94–96) and peripheral terminals (97). In this study, LC secretome profiling revealed increased CCL2 release from HFD LCs under inflammatory challenge, aligning with scRNA-seq evidence of chemokine program upregulation. Moreover, local CCR2 antagonism produced a rapid, time-limited reversal of mechanical allodynia, consistent with an ongoing requirement for peripheral CCR2 signaling to maintain nociceptor sensitization in this model. These findings support a model in which HFD induces LC activation and chemokine release that acts locally at epidermal terminals and/or via DRG pathways to enhance nociceptor responsiveness, thereby promoting PDN pain.

Our findings align with and extend growing evidence that nonneuronal cells within the skin contribute to PDN pathogenesis (18, 19). While diabetes is a multifactorial disease, immune-mediated mechanisms are likely important contributing factors (19). Chronic low-grade inflammation is a defining feature of diabetes, with elevated circulating and tissue levels of IL-6, IL-2, and TNFA reported in patients (21–23), and increased dermal macrophage density observed in painful compared with nonpainful diabetic neuropathy (98). Activated LCs are known to secrete proinflammatory cytokines and mediators that can influence nociceptor excitability (18). Using high-multiplex cytokine profiling, we identified a selective inflammatory secretory profile in HFD-exposed LCs, characterized by upregulation of MCP-1/CCL2. This finding is particularly relevant given extensive evidence that CCL2-CCR2 signaling promotes pain hypersensitivity in naive rodents and contributes to neuropathic pain following nerve injury (87–93). Genetic deletion or pharmacological inhibition of CCR2 abolishes pain hypersensitivity in multiple preclinical models (85, 89). Our data extend this framework by identifying LCs as a direct source of pronociceptive chemokines, such as CCL2, which is known to modulate DRG neuron excitability (99) via receptors expressed on the cell body (74, 94–96) and on terminal afferents (97). Moreover, we demonstrated that topical intraplantar blockade of CCL2-CCR2 signaling significantly attenuates mechanical allodynia in HFD-fed mice, supporting a functional role for LC-derived CCL2 in PDN-associated nociceptor sensitization. Importantly, increased CCL2 levels have been reported in both diabetes and obesity in humans (100–102). CCL2 expression is elevated in circulating monocytes from patients with type 1 diabetes, and serum CCL2 levels are increased in type 2 diabetes, where they correlate with insulin resistance and BMI (100–104). Indirect contributions from CCR2^+^ myeloid cells cannot be fully excluded and represent a limitation of this study as well as an important area for future investigation. Nevertheless, our findings extend prior observations by identifying epidermal LCs as a local cutaneous source of CCL2 in PDN, thereby providing a mechanistic link between metabolic inflammation, skin-resident immune activation, and peripheral sensory dysfunction.

Beyond chemokines, our single-cell and interactome analyses suggested additional candidate mechanisms for LC-nociceptor communication. In males, HFD LCs upregulated axon-guidance receptors, including *Plxnb2* and *Plxna1*, and ligand-receptor inference suggested semaphorin-plexin signaling between LC populations and non-peptidergic/Mrgprd-positive nociceptors. Semaphorin-plexin pathways are established regulators of axonal patterning and structural plasticity (105–107) and modulate immune cell activation and migration (104–110). The upregulation of *Plxnb2* in LCs is notable given prior reports implicating this receptor in microglia and macrophages following nerve injury, where it contributes to pain behavior (108, 109). Thus, semaphorin-plexin signaling may couple immune activation to changes in epidermal neuroimmune architecture that favor sensitization. However, these computational inferences are inherently correlative; definitive testing will require LC-specific genetic perturbation and spatially resolved validation of pathway engagement at the epidermal nerve interface.

A long-standing paradox in PDN is the coexistence of increased pain sensitivity with loss of intraepidermal nerve fibers (IENFs) (3, 5, 54). Although reduced IENF density is widely used to diagnose small fiber neuropathy, it does not reliably distinguish painful from nonpainful diabetic neuropathy (98). Increasing evidence indicates that PDN-associated cutaneous remodeling involves dynamic degeneration and regeneration of specific nociceptor subtypes (108–111). In prior work, we showed that HFD feeding induces progressive loss of Nav1.8-positive fibers while sparing the specific subtype of the *Mrgprd*-positive fibers (57). In the present study, to test whether LC-dependent behavioral effects could be explained by altered cutaneous innervation, we generated and analyzed a *Mrgprd* eGFP–hLCDTR mouse line. In this model, CD207^+^ cell depletion again prevented HFD-induced mechanical allodynia yet did not significantly alter *Mrgprd*^+^ intraepidermal nerve fiber density at the analyzed disease stage (Supplemental Figure 3). Similarly, quantification of total PGP9.5^+^ fibers revealed only modest, nonsignificant changes following CD207^+^ cell depletion (Supplemental Figure 3). These findings indicate that CD207^+^ cell-dependent pain behaviors cannot be attributed to gross loss of *Mrgprd*^+^ fibers and support the interpretation that immune-driven sensitization can occur independently of measurable changes in fiber density. This is consistent with the broader view that PDN involves dynamic remodeling and functional dysregulation of specific nociceptor subtypes rather than denervation alone (108–111). Fully resolving this paradox will require dedicated analyses of subtype-specific remodeling and functional connectivity across disease stages.

An additional consideration is the identity and heterogeneity of CD207^+^ epidermal cells. Recent studies have raised the possibility that epidermis contains transcriptionally distinct LC subsets that could not correspond to traditional LCs (42, 110–112). Our scRNA-seq analysis identified multiple CD207^+^ subclusters, including a *Ly6g6c*-expressing population consistent with LC heterogeneity and the potential presence of monocyte-derived LC-like cells under inflammatory conditions. However, given the ongoing debate regarding whether some CD207^+^ epidermal populations represent bona fide LCs or distinct dendritic cell lineages (42, 110–112), we avoid definitive ontogenetic assignments for these subsets. Species-specific differences in epidermal immune composition and plasticity may also contribute to differences between mouse and human observations, particularly regarding LC density changes.

To enhance the translational relevance of our observations, we analyzed skin biopsies from patients with clinically characterized PDN. While LC density was not significantly altered compared with controls, 3D morphometric analysis revealed increased LC volume and dendritic complexity that correlated with diabetes duration, consistent with progressive LC remodeling in chronic disease. Because the onset of neuropathy is often difficult to define retrospectively, duration of diabetes provides a more reliable temporal reference in this cohort. We avoided causal interpretation of the reduced IENFD/LC ratio observed in PDN skin, as this metric can reflect decreased innervation in the context of stable LC density. Instead, our human biopsy analyses provide translational relevance by identifying LC morphological remodeling that correlates with disease duration in PDN. Because LC morphology is linked to activation and functional state (51, 113), increased arborization and cell volume likely reflect chronic stimulation in diabetic skin. However, interpretation is limited by cohort size, clinical heterogeneity — including variability in pain severity and sensory phenotypes (113) — and the cross-sectional nature of the sampling, which precludes causal inference. Longitudinal studies and stratification by sensory phenotype, pain severity, and metabolic control will be important to determine whether LC remodeling predicts pain trajectories or treatment response and whether LC-derived chemokines associate with clinical outcomes.

In summary, our studies have identified epidermal LCs as essential regulators of neuropathic pain behavior in male PDN through chemokine-dependent neuroimmune signaling and reveal sex differences in LC contribution to disease. By integrating functional genetics, single-cell transcriptomics, computational interactomes, and pharmacological validation, we provide convergent evidence that skin-resident immune cells can shape nociceptor dysfunction independently of overt nerve loss. The superficial localization and accessibility of LCs, together with the efficacy of local CCR2 blockade, highlight the skin as a promising target for peripherally acting, immunomodulatory strategies for PDN and other painful neuropathies that might share similar mechanisms. More broadly, these findings support a framework in which immune cells within barrier tissues can actively instruct chronic pain states.

## Methods

### Sex as a biological variable.

Both male and female mice were included, and human biopsy data included both male and female patients with PDN and sex-matched individuals who were healthy controls. Sex was considered a biological variable, and sex-dependent differences were identified and analyzed.

### Animals.

Animals were housed on a 12-hour light/12-hour dark cycle with ad libitum access to food and water. Adult wild-type male and female mice between 6 and 8 weeks were adopted for most of the experiments. Both male and female were fed a high-fat diet (HFD 42% fat - EnvigoTD88137, Envigo, Madison, WI) or a regular diet (RD 11% fat) for 10 weeks and then a glucose tolerance test was performed as first described in Menichella et al. 2016 (41).

### Skin biopsies.

All subjects underwent clinical examination, skin biopsy and gave informed consent to participate in the study. For the present study, 15 patients with diabetic neuropathy (7 females and 8 males) and 9 healthy controls (4 females and 5 males) were enrolled. Clinical features were collected using an established protocol previously described (114). Pain intensity was measured as the average score of the last 3 weeks using the pain intensity numerical rating scale (PI-NRS).

Expanded methods and further information may be found in Supplemental Materials.

### Statistics.

All statistical analyses was performed using R studio (2023.12.0+369) or GraphPad Prism (10.0.3). A Shapiro-Wilk test was applied to assess normality. For comparisons between 2 groups, a 2-tailed student’s *t* test was applied, and, where applicable, adjustments for multiple testing were made and reported in figure legends. For comparisons involving more than 2 groups, 2-sided 1-way or 2-way ANOVA was performed, followed by post-hoc multiple comparison testing, as reported in figures legends. For longitudinal behavioral data involving repeated measurements within the same animal or cell condition, repeated measures ANOVA was applied. Pearson correlation analysis was performed to assess the correlation between variables and the correlation coefficient (*r*) and p value were reported within the graphs. Quantification of LCs density, RNAscope, and behavioral tests were performed in double-blind manner. All values are expressed as the mean ± SEM, and a *P* value of less than 0.05 was considered statistically significant.

### Study approval.

This research complies with all relevant ethical regulations.

All animal care protocols and experiments were reviewed and approved by the Institutional Animal Care and Use Committee (IACUC) of Northwestern University.

Human tissues were collected from healthy volunteers and patients and the study was approved by the local Ethical Committee of the Fondazione IRCCS Istituto Neurologico ‘Carlo Besta’ of Milan (FINCB), Italy. Additional information may be found in Supplemental Materials.

### Data availability.

All data presented in this study are included in the main text and Supplemental figures. The scRNA-seq data generated from paw epidermis of male and female RD and HFD mice have been deposited in NCBI’s Gene Expression Omnibus (GEO) and are accessible through GEO Series accession number GSE327170 (https://www.ncbi.nlm.nih.gov/geo/query/acc.cgi?acc=GSE327170). All data underlying the graphs are provided in the Supporting Data Values file.

## Author contributions

PP performed scRNA-seq data analysis, pain behavioral studies, immunofluorescence staining of whole-mount and skin sections, LCs’ isolation, sorting and cytokine assay, statistical analysis and figures. DG performed scRNA-seq of paw epidermis. DNDJ performed von Frey behavioral studies, testing for diabetes, RNAscope in situ hybridization, LCs quantification. DR performed epidermal cell cultures, mouse breeding and administration of HFD. JSCD performed epidermal cell cultures. SV performed supplemental transcriptomic data analysis. AAB and ASP assisted with feedback on the manuscript. MA, RL, and DC performed the immunofluorescence staining and analysis of human skin samples. DC performed the morphometric analysis. GD enrolled and characterized PDN patients and controls. GLP supervised the analysis of human skin data. DMM supervised the project and provided fundings. DMM and PP drafted and edited the manuscript. DMM and RJM reviewed the manuscript. All authors read and approved the manuscript.

## Conflict of interest

The authors have declared that no conflict of interest exists.

## Funding support

This work is the result of NIH funding, in whole or in part, and is subject to the NIH Public Access Policy. Through acceptance of this federal funding, the NIH has been given a right to make the work publicly available in PubMed Central.

- NIH R01 NS104295-01
- NIH HEAL initiative supplement R01 NS104295-01 (DMM),
- NIH R01 AR077691-01 (DMM and RJM).

## Supplementary Material

Supplemental data

Supporting data values

## Figures and Tables

**Table 1:**
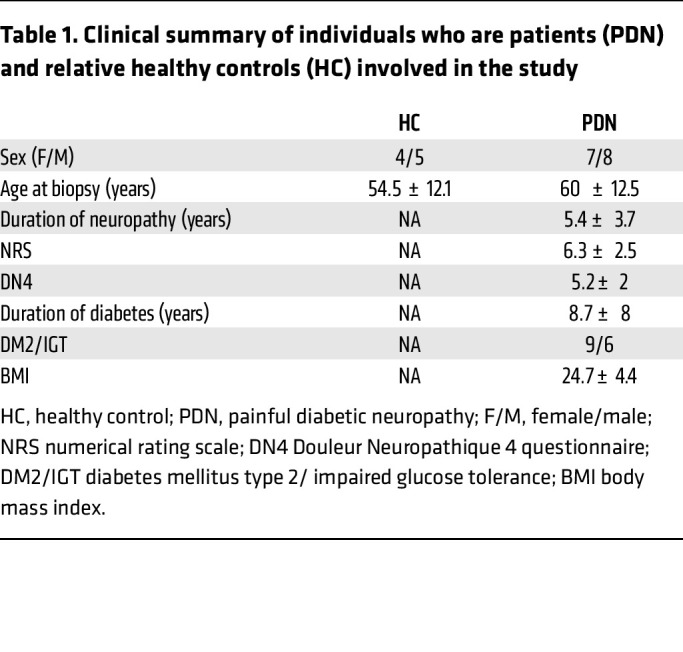
Clinical summary of individuals who are patients (PDN) and relative healthy controls (HC) involved in the study

## Version 1 04/30/2026

In-Press Preview

## Version 2 06/15/2026

Electronic publication
