# Supplementary material for: Skin-resident Langerhans cells drive neuropathic pain via chemokine-dependent neuron-immune communication
Source: J Clin Invest. 2026 Apr 30;136(12):e192328. doi: 10.1172/JCI192328 (PMC13262737; doi:10.1172/JCI192328)
Supplement: Supplemental data [file jci-136-192328-s224.pdf]

Skin-Resident Langerhans Cells Drive Neuropathic Pain via Chemokine-Dependent Neuron–Immune Communication.

[1]Paola Pacifico, [2]Dale George, [1]Nirupa D. Jayaraj, [3]Dongjun Ren, [1]James S. Coy-Dibley, [3]Abdelhak A. Belmadani, [1]Sofia Veronesi, [4]Mirna Andelic, [4]Daniele Cartelli, [4]Grazia Devigili, [4]Raffaella Lombardi, [4,5] Giuseppe Lauria Pinter, [6]Amy S. Paller, [3]Richard J. Miller, [1,3*]Daniela M. Menichella.

[1]Department of Neurology, Feinberg School of Medicine, Northwestern University, Chicago, IL.
[2]SonoThera, Inc., South San Francisco, CA 94080
[3]Department of Pharmacology, Feinberg School of Medicine, Northwestern University, Chicago, IL.
[4]Neuroalgology Unit, Fondazione IRCCS Istituto Neurologico Carlo Besta, Milan, Italy
[5]Department of Medical Biotechnology and Translational Medicine, University of Milan, Milan, Italy.
[6]Department of Dermatology, Feinberg School of Medicine, Northwestern University, Chicago, IL.

*Address correspondence to: Daniela Maria Menichella, Department of Neurology and Pharmacology, Feinberg School of Medicine, Northwestern University, Lurie 8-123, 303 E. Superior Street, Chicago, Illinois 60611, USA. Email: d-menichella@northwestern.edu. Phone: +1 312-503-3223

All authors declare that they have no conflicting interests.

SUPPLEMENTAL MATERIALS

METHODS

Sex as a biological variable

Both male and female mice were included, and human biopsy data included both male and female PDN patients and sex-matched healthy controls. Sex was considered a biological variable, and sex-dependent differences were identified and analyzed.

Animals

Animals were housed on a 12-hour light/12-hour dark cycle with ad libitum access to food and water. Adult wild-type male and female mice between 6 and 8 weeks were adopted for most of the experiments. Both male and female were fed a high-fat diet (HFD 42% fat - EnvigoTD88137, Envigo, Madison, WI) or a regular diet (RD 11% fat) for 10 weeks and then a glucose tolerance test (GTT) was performed as first described in Menichella et al. 2016(1). After fasting, mice were injected with a 45% D-glucose solution (2mg glucose/g body weight) and blood glucose was measured at 30, 60, and 120 minutes after injection. To compare "diabetic" versus "non-diabetic" HFD mice, we set the cutoff for diabetes ≥140 mg/dl for male mice(2) and ≥130 mg/dl for female mice at 2 SD above the mean for glucose 120 minutes after glucose challenge. In addition to wild-type mice (C57BL/6J), we used the humanized Langerin-diphtheria toxin receptor mouse line(3) (h*Langerin*-DTR - C57BL/6J), kindly provided by D. Kaplan at Pittsburg University, and the reporter Nav1.8-dtTomato mouse line, previously described in Jayaraj et al., 2018(2). Additionally, we generated a double transgenic mouse line by crossing C57BL/6J *Mrgprd*-eGFP mouse line(4) with hLC-DTR mice (*Mrgprd*eGFP-hLCDTR)

Skin biopsies

All subjects underwent clinical examination, skin biopsy and gave informed consent to participate in the study. For the present study, 15 patients with diabetic neuropathy (7 females and 8 males) and 9 healthy controls (4 females and 5 males) were enrolled. Clinical features were collected using an established protocol previously described(5). Pain intensity was measured as the average score of the last 3 weeks using the pain intensity numerical rating scale (PI-NRS).

Single-cell dissociation from paw epidermis

Glabrous hind paw skin was harvested from the mouse and incubated in Dispase (2.3 mg/ml, Sigma-Aldrich D4693) overnight at 4C. The following day, the epidermis was gently peeled off from the dermis and incubated in TrypLE Express (Gibco 12604-013) for 10 minutes at 37C. Single cells were dissociated using gentle agitation, filtered with a 30 μm cell strainer into a sterile tube and then resuspended in 154CF epidermal medium (Gibco M154CF500) or in RPMI 1640 (Gibco 11875093)

Cell sorting and primary culture of epidermal LCs.

Single cells isolated from the paw epidermis and collected in RPMI 1640 were centrifuged at 300g for 5 minutes at 4C. The cell pellet was resuspended in FACS buffer (1% BSA in 1xPBS). LCs were selectively labeled using PE anti-human CD207 1:100 (Biolegend, 352203) and APC andi-mouse CD45 1:100 (Biolegend, 103111) for 2hours at 4C in the dark. After staining, the single cells suspension was washed three times with 1xPBS and resuspended in fresh 1XPBS. Given the fragile nature of LCs, the MACSQuant® Tyto® Cell Sorter was used to sort and collect double-positive cells. Aseptic cartridges were

employed to load the cells, and only double positive cells CD207$^+$/CD45$^+$ were collected in the middle compartment of the cartridge and subsequently recovered.

The sorted CD207$^+$/CD45$^+$ LCs were resuspended in RPMI 1640 enriched with L-glutamine and 2-Mercaptoethanol (2-ME) and plated onto IgG-coated surfaces(6). The plated cells were then stimulated with recombinant mouse TNFA (R&D, 410-MT) at a final concentration of 125U/mL and recombinant mouse GM-CSF (R&D, 415-ML) at a final concentration of 500U/mL. 50 μL of medium from both stimulated and un-stimulated LCs derived from RD and HFD mice was collected at various time point and stored at -80C for subsequent analysis.

Immunohistochemistry

Whole-mount staining. Glabrous hind paw skin was harvested from the mouse and incubated in Dispase (2.3 mg/ml, Sigma-Aldrich D4693) overnight at 4C. The following day, the epidermis was gently separated from the dermis. The isolated paw epidermis was fixed with 4% PFA for 30 minutes at room temperature. After fixation, tissues were washed thoroughly with 1XPBS, and the whole tissue was divided in smaller pieces. Only the central area of the hind paw including footpads was processed for histological analysis. The tissue was first permeabilized with 0.2% Triton X-100 in PBS for 15 minutes, and then incubated in a blocking solution containing 10% NDS - 0.1% BSA in 0.1% Triton X-100-PBS for 1hour at room temperature. The tissue was then incubated with APC anti-mouse/human Cd207 1:100 (Biolegend, 4C7) overnight at 4C and images were acquired using a confocal fluorescence microscope.

Skin sections. Glabrous hind paw skin was harvested from the mouse, fixed with 4% PFA for 90 minutes at room temperature, and then stored in 30% sucrose overnight at

4C. The following day, tissue was included in O.C.T. and stored at -80C. 20um cryosections, placed on superfrost plus slides (Thermo Fisher Scientific), were stained with anti-K14 1:500 (906004, Biolegend), anti-PGP9.5 1:100 (SAB4503057), anti-K10 1:500 (Ep1607ihcy, Abcam) and anti-CD207 1:100 (4C7, Biolegend). Secondary antibodies Alexa Fluor 488 goat anti-rabbit antibody (Invitrogen, Thermo Fisher Scientific, 1:250), was used.

Human skin sections. Skin biopsy samples were taken from the distal leg (10 cm above the lateral malleolus) following the standard procedure(7). Specimens were then fixed in 2% PLP, cryoprotected and cut into 50 um vertical sections. Samples were washed twice in PBS, then kept for an hour in blocking solution (1%BSA + 2% NGS + 0.5% Triton X-100 in PBS). Incubation with mouse anti-CD207 conjugated to Alexa 633 (Biolegend, 1:100) and rabbit anti-PGP9.5 (Proteintech, 1:1500) was performed overnight on room temperature. After two washes in PBS, secondary antibody incubation Goat-Anti-Rabbit 488 (1:2000) was carried out for 1h on RT. Samples were examined with a confocal laser scan microscope (TCS SP8 AOBS; Leica Microsystems) equipped with Ar/Ar-Kr 488 and 633 nm diode lasers. 3 random images were acquired for each sample by using a HC PL APO CS 40x/1.1 Water immersion objective and HyD detectors. Laser intensity and photo multiplier gain for each channel was adjusted to minimize background noise and saturated pixels, and once defined for control conditions, parameters were kept constants for all acquisitions.

To measure the density of fibers innervating the epidermis, intraepidermal nerve fiber (IENF) density, expressed as the number of nerves crossing the epidermal-dermal junction as a function of length(2) was measured.

RNAscope in situ hybridization

RNAscope in situ hybridization multiplex V2 was performed according to the manufacturer's instructions, Advanced Cell Diagnostics (ACD). Twelve-micrometer DRG cryosections from RD and HFD male mice were incubated with probes *Mrgprd* (417921-C2/C3), *Sema4d* (498381-C3), *Sema6d* (565871-C1). More details reported in George et al., 2024(8).

Analysis. DRG sections were analyzed by imaging the whole DRG using Olympus FV10i confocal microscope, and the images were processed using Fiji. Target mRNA expression was measured as average intensity of target dots per cell(8). First the average background intensity (ABI) based on the integrated intensity of a background region in a selected area (ABI5IntDen background/ area of selected background) was calculated. Then, 10 dots per cell (3 cells per section) were considered and the area and integrated intensity of each dot was measured. Lastly, the average intensity per single dot (AISD) was calculated using the formula: AISD = IntDen selected dots - ABI x area of selected dots / tot number of dots. Mean values of the counts from blinded reviewers were plotted and graphed.

Morphometric analysis

Images were processed with FIJI and Arivis Pro (rel 4.2). By using the Neurite Tracer pipeline of Arivis Pro we determined the total number of branching points and the volume occupied by LCs. The parameters used in our set up are:

- Method: Probabilistic Reconstructor
- Branch diameter: 0.284-5 um
- Tubularity sensitivity: 12

- Seeding-Tubularity local threshold: 0.2/ Seed Filter: 40%
- Min Terminal section length: 2 mm

Behavioral test

Mechanical allodynia test (von Frey) To assess mechanical allodynia, the von Frey test was performed as follow. RD and HFD male and female mice were placed on a metal mesh floor under a transparent plastic dome. Following 60-min of habituation, seven different filaments, each with a specific bending force (10, 20, 40, 60, 80, 100, and 120 mN) were applied in ascending order to the plantar surface of the hind paw. Each filament was applied six times with an interstimulus interval between 10-15 seconds. The von Frey withdrawal threshold was defined as the minimum force eliciting a detectable withdrawal response in at least 50% of trials.

Spontaneous pain test (cage-lid hanging) To test spontaneous pain, the cage-lid hanging test was adapted from Zhang et al., 2021(9). Briefly, mice were acclimated to the behavioral testing room for 30 minutes before the test. An empty polypropylene cage (dimension: 290x220x140mm) with a slightly modified lid was used for the test. Each mouse was placed on the underside of the inverted lid, and the time to fall was recorded. The test was repeated three times for each mouse with a maximum cutoff time of 30 seconds.

Researchers conducting behavioral tests and endpoint analyses were blinded to the experimental conditions.

Diphtheria toxin-mediated ablation. To deplete LCs and maintain their depletion throughout the critical window during which mechanical allodynia normally develops in

HFD male mice (2), hLC-DTR mice received weekly injections of Diphtheria toxin (DT 4ng/gr of body weight) from week 10 to week 14 of age.

Control mice were administered vehicle (0.9% NaCl). Paw epidermis was collected 48h after the last injection and the efficiency of LC ablation was assessed by quantifying CD207+ LCs in epidermal whole-mount preparation.

scRNAseq

Sample processing and raw data analysis

For the scRNAseq of paw epidermis of three adult RD and HFD male and female mice, we generated a single-cell suspensions as described in "*Single-cell dissociation from paw epidermis*" paragraph. We assessed cell viability using an automated cell counter and for all samples we achieved >90% cell viability.

For the sequencing, we choose the droplet-based method by 10X Genomics where each droplet should contain a single cell. Libraries were prepared by NUSeq Core at Northwestern University according to 10X Genomics instructions. Sequencing was performed on an Illumina platform, and FASTQ raw sequencing data were aligned to mouse transcriptome reference using Cell Ranger toolkit from 10X Genomics. The downstream data analysis and visualization was performed using Seurat 5 pipeline in R.

Quality control, clustering, and differential gene expression analysis The standard pre-processing workflow for scRNAseq data in Seurat 5 (version 5.0.0, https://satijalab.org/seurat/)(10) was performed following QC metrics. To remove low-quality cells, potential doublets, or dying cells, filter parameters were applied to both male and female datasets. For paw epidermis of male sample datasets, cells with <200 and >6000 genes, and >5% mitochondrial genes detected were excluded. For paw

epidermis of female sample datasets, cells with <500 and >6000 genes, and >5% mitochondrial genes detected were removed. Seurat 5 in R was used to separately analyze male and female datasets. Features expressions were normalized using Log-normalize and high variable features were identified by FindVariableFeatures function of Seurat package. Principal components analysis was performed using RunPCA, and the top 10 principal components, identified by the cumulative variance analysis, were used for clustering. Uniform Manifold Approximation and Projection (UMAP) was run for clusters' visualization. Differentially expressed genes between RD and HFD groups were identified using a Wilcoxon Rank Sum test, corrected for false discovery rate (FDR), and visualized using the EnhancedVolcano R package. Only genes with log2fold change above 0.25 were considered. The analysis to identify pathways and gene set enrichment was performed using Gene Set Enrichment Analysis (GSEA). Data were visualized using R packages: Seurat, ggplot2, dittoSeq.

scRNAseq data integration scRNAseq datasets from male and female paw epidermis both RD and HFD were integrated using anchor-based CCA integration that corrects for batch effects across sexes and diet conditions, thereby improving comparability between samples. A total of 12 samples including 6 males and 6 females were analyzed.

Cell-cell interaction data analysis To predict intercellular communication, CellChat (v1), a package developed in R(11), was applied to male scRNAseq dataset. CellChat is designed for inference, analysis, and visualization of cell-cell communication from scRNAseq data. The CellChat object created from the Seurat object was aligned to mouse ligand-receptor database to create annotations. The ligand-receptor CellChatDB is based on KEGG (Kyoto Encyclopedia of Genes and Genomes).

"computeCommunProb" function was adopted to infer cell-cell communications within the dataset and then standard workflow for visualization was applied to generate graphs.

Interactome analysis platform To infer ligand-receptor-mediated signaling between DRG neurons and LCs the interactome analysis platform(12) was adopted. Male NP1 DRG cluster isolated from the DRG HFD and RD scRNAseq dataset(4) was integrated to male LC from epidermis HFD and RD scRNAseq dataset. The interaction between the clusters was analyzed using the average expression of each cluster and matched with a database of known ligand-receptor. Plots were generated using SankeyMatic.

To infer intercellular communication between LCs and DRG, DRG scRNAseq dataset from RD and HFD male mice was used. Raw matrix data are deposited in Dryad (https://doi.org/10.5061/dryad.9s4mw6mm5) and/or available upon request(4).

Multiplex cytokine analysis

The cytokine profile of LCs was assessed using the Codeplex Secretome platform by Isoxplexis (Bruker, Cellular Analysis) designed to the molecules of mouse innate immune response. Medium collected from primary LCs culture, as described in the *"Cell sorting and primary culture of epidermal LCs",* was loaded onto Isoplexis chip (CODEPLEX-2L12-1) for secretome analysis. Cytokines levels were detected and represented as as Relative Fluorescence Unit (RFU) and reported either as Log-Transformed values or pg/mL. The analysis was conducted with the technical support of Bruker specialists.

Statistics

All statistical analyses was performed using R studio (2023.12.0+369) or GraphPad Prism (10.0.3). A Shapiro-Wilk test was applied to assess normality. For comparisons between

two groups, a two-tailed student's t-test was applied, and where applicable, adjustments for multiple testing were made and reported in figure legends. For comparisons involving more than two groups, one-way or two-way Analysis of Variance (ANOVA) two-sided was performed, followed by post-hoc multiple comparison testing, as reported in figures legends. For longitudinal behavioral data involving repeated measurements within the same animal or cell condition, repeated measures ANOVA was applied. Pearson correlation analysis was performed to assess the correlation between variables and the correlation coefficient (*r*) and p value were reported within the graphs. Quantification of LCs density, RNAscope and behavioral tests were performed in double-blind manner. All values are expressed as the mean ± SEM, and a *P* value of less than 0.05 was considered statistically significant.

Study approval

This research complies with all relevant ethical regulations.

All animal care protocols and experiments were reviewed and approved by the Institutional Animal Care and Use Committee (IACUC) of Northwestern University.

Human tissues were collected from healthy volunteers and patients and the study was approved by the local Ethical Committee of the Fondazione IRCCS Istituto Neurologico 'Carlo Besta' of Milan (FINCB), Italy.

All procedures were designed to maximize robustness and minimize bias. Experimenters performed and analyzed LCs density in whole-mount epidermal sheets, RNAscope and behavioral assays were blinded to diet treatment (RD or HFD) and/or compound (diphtheria toxin or vehicle) until data analysis is complete. Both male and female mice were included in all experiments and results were analyzed separately and compared.

To collect human tissues from PDN patients and controls, we first performed clinical examination of all subjects and obtained informed consent to participate in the study. For the present study, 15 patients with diabetic neuropathy (7 females and 8 males) and 9 healthy controls (4 females and 5 males) were enrolled. Clinical features were collected using an established protocol previously described(5). Pain intensity was measured as the average score of the last 3 weeks using the pain intensity numerical rating scale (PI-NRS). Further information as number of animals used, replications, number of sections counted are indicated in the figures and/or figure legends. On the graphs, individual dots represent individual samples/mice used.

SUPPLEMENTAL FIGURES

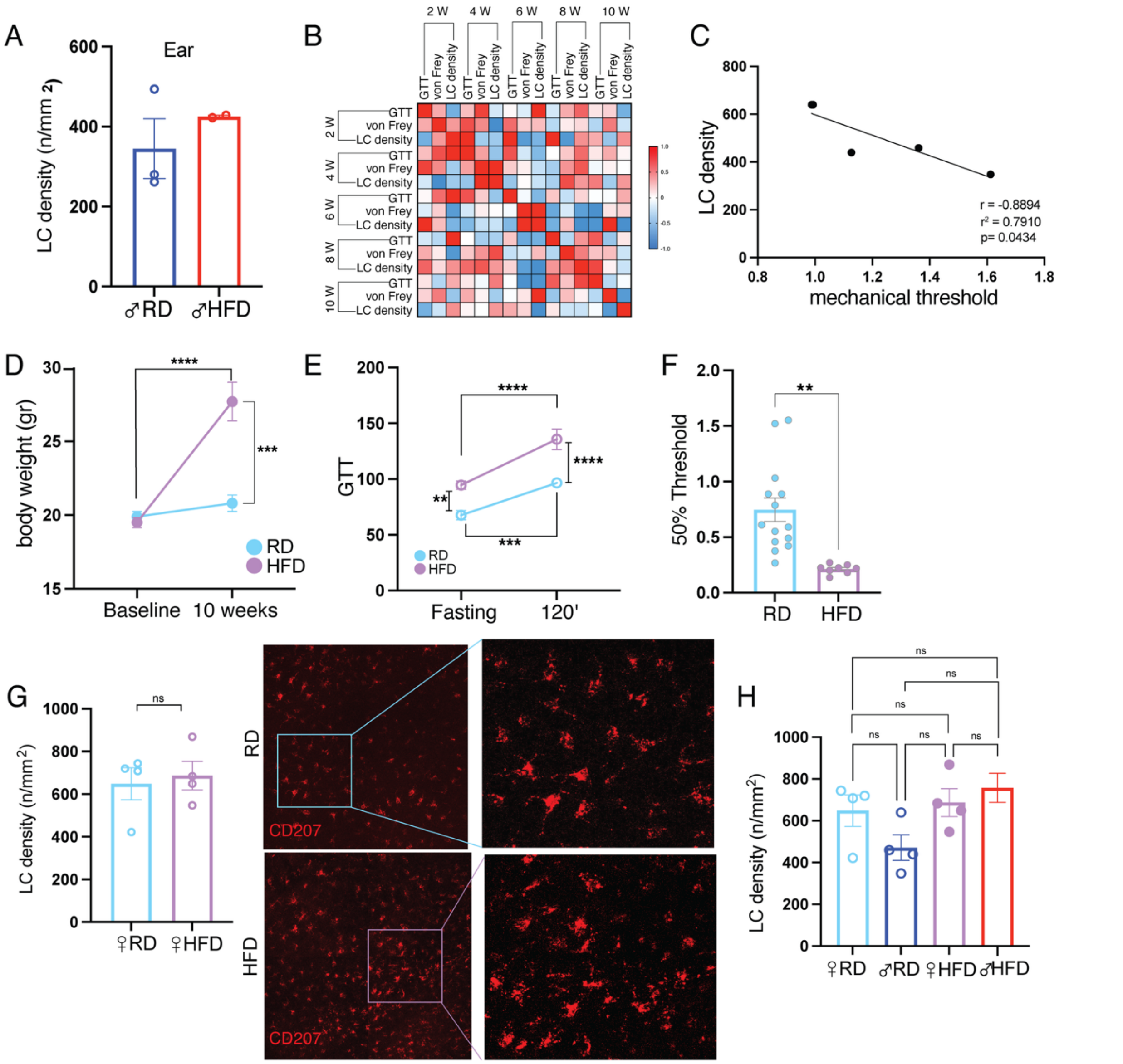

Supplemental Figure 1. LCs density in RD mice (A) LCs density measured as numb of cells/area (mm^2) in the ear of male mice. No differences between RD and HFD male animals. Unpaired two tailed t-test with Welch's correction p=0.3940. RD n=3 and HFD n=2. **(B)** Correlation matrix of RD features (GTT, von Frey and LCs) at different time points. **(C)** Negative correlation between LCs density and von Frey in RD male mice. Pearson r coefficient r=-0.8894, r^2=0.7910, p= 0.0434(*). **(D)** Body weight (gr) in female mice. n=10 animals per each diet conditions. Two-way ANOVA for multiple comparison. Between RD and HFD: 10 weeks p=0.0002 (***). Within HFD: baseline vs 10weeks p<0.0001. **(E)** Glucose tolerance test (GTT) in female mice. RD n=14 and HFD n=12. Two-way ANOVA for multiple comparison. Between RD and HFD: Fasting p=0.0011 (**); 120' p<0.0001 (****). Within RD: Fasting vs 120' p=0.0003 (***). Within HFD Fasting vs 120' p<0.0001(****). **(F)** von Frey test. RD n=14 and HFD n=8. Unpaired two-tailed t-Test p=0.0014 (**). **(G)** Quantification of LCs' density (number of LCs / mm^2) in RD and HFD female mice at 10 weeks (b) and representative images (c). n=3 sections per animal were acquired and Cd207+ cells per 0.04 mm^2 per section area were counted. Unpaired two-tailed t-Test p=0.7154. n=4 for both groups. **(H)** LCs' density

compared among all groups. One-way ANOVA for multiple comparisons. RD female vs. RD male p=ns; HFD female vs. HFD male p=ns; RD female vs. HFD female p=ns; RD female vs. HFD male p=ns; RD male vs. HFD male p=0.045 (*); RD male vs HFD female p=ns.

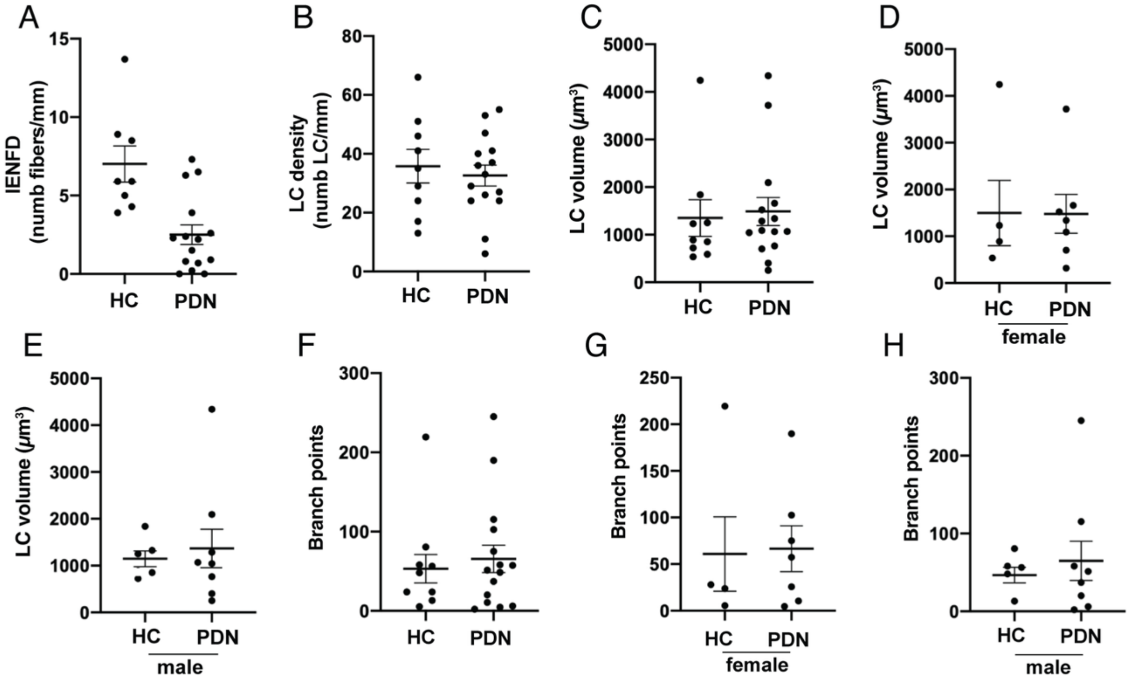

Supplemental Figure 2. **LCs features analysis in PDN patients (A-B)** Plots of the intraepidermal nerve fiber density (IENFD) and the LCs density in the entire cohort of healthy subjects (HC) or diabetic patients (PDN). **(C-E)** LCs volume expressed in μm^3 in the (c) entire cohort, (d) in female or (e) in male subjects. **(F-H)** branch points of LCs in in the (f) entire cohort, (g) in female or (h) in male subjects. No differences between HC and PDN. Middle lines of the plots represent the mean value, while whiskers are SEM

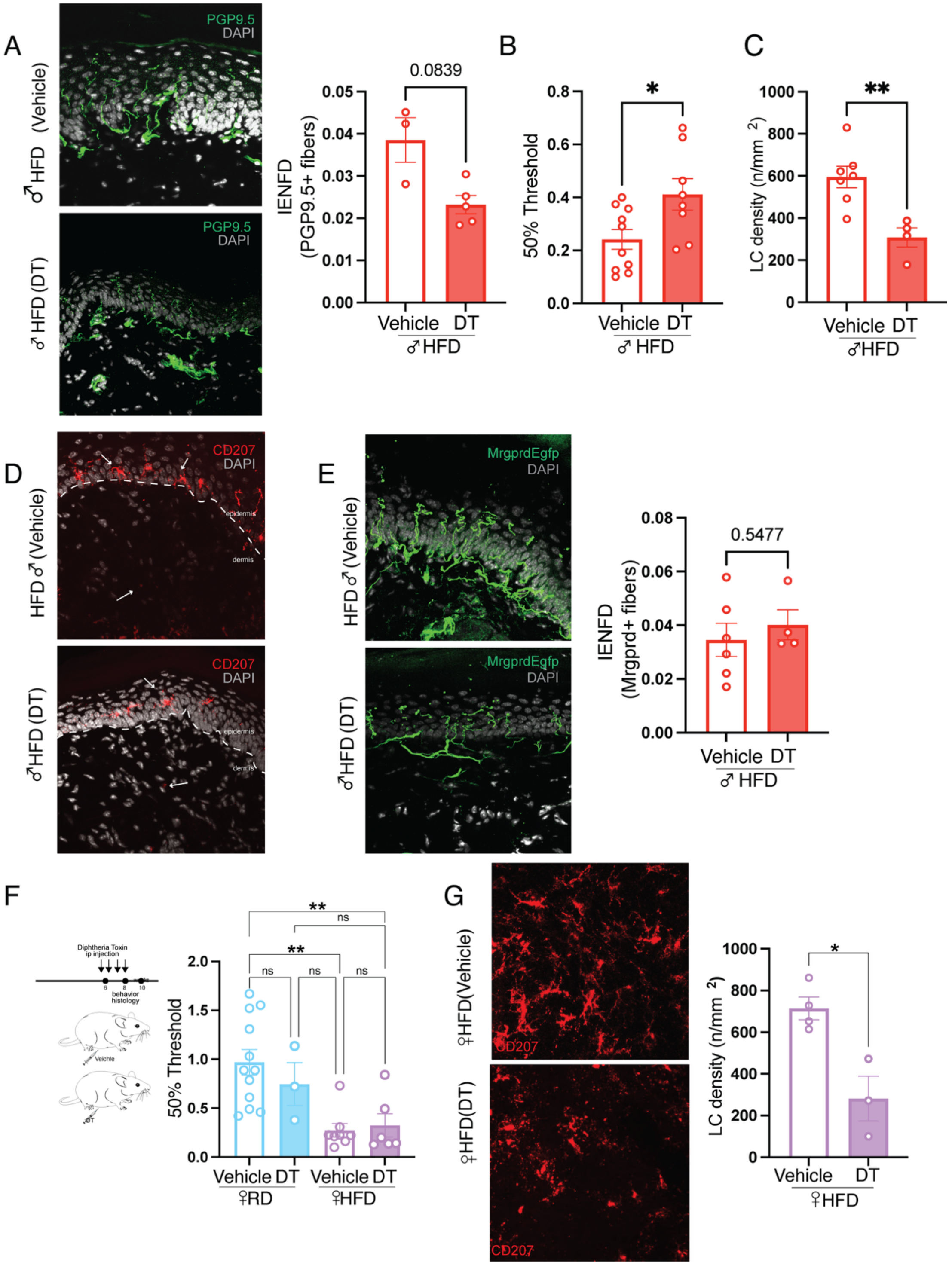

Supplemental Figure 3. IENFD LC-mediated (A) IENFD of PGP9.5-positive fibers in HFD DT-ablated and HFD control mice. Quantification of PGP9.5 IENFD. Unpaired t-test with Welch's correction p= 0.0839. Three random areas per each animal were analyzed. Two counters. HFD (vehicle): n= 3 HFD (DT): n=5. **(B)** Response to evoked mechanical stimuli measured in von Frey test in HFD DT- and vehicle-treated MrgprdeGFP-hLCDTR double transgenic mice**.** Unpaired t-test with Welch's collection. p=0.0324 (*). HFD (vehicle) n=10, HFD (DT) n=8**. (C)** LC density measured as the numb of Cd207+ cells per area in MrgprdeGFP-hLCDTR**.** Unpaired t test with Welch's correction p= 0.0026 (**). HFD (vehicle) n=6, HFD (DT) n=4. **(D)** Selectivity of DT-mediated LC depletion. Dashed line indicates the separation between epidermis

and dermis**.** **(E)** IENFD of Mrgprd-eGFP-positive fibers in MrgprdeGFP-hLCDTR. Unpaired t-test p=0.5477. HFD (vehicle) n=7, HFD (DT) n=4. **(F)** cartoon of LC DT-mediated ablation in female mice and Response to evoked mechanical stimuli threshold through von Frey test. DT-ablation does not recover mechanical allodynia in HFD female. One-way ANOVA for multiple comparison. RD (vehicle) vs HFD (vehicle) p=0.0013 (**), RD (vehicle) vs HFD (DT) p=0.0068 (**). RD (vehicle): n= 14, RD (DT): n=3, HFD (vehicle) n=7; HFD (DT) n=6. **(G)** LC density measured as the numb of Cd207+ cells per area**.** Unpaired t test with Welch's correction p= 0.0366. Representative confocal images of paw epidermal sheets of HFD female mice injected with vehicle (0.9% NaCl) or DT 4ng/gr body weight. HFD (vehicle) n=4, HFD (DT) n=3.

355

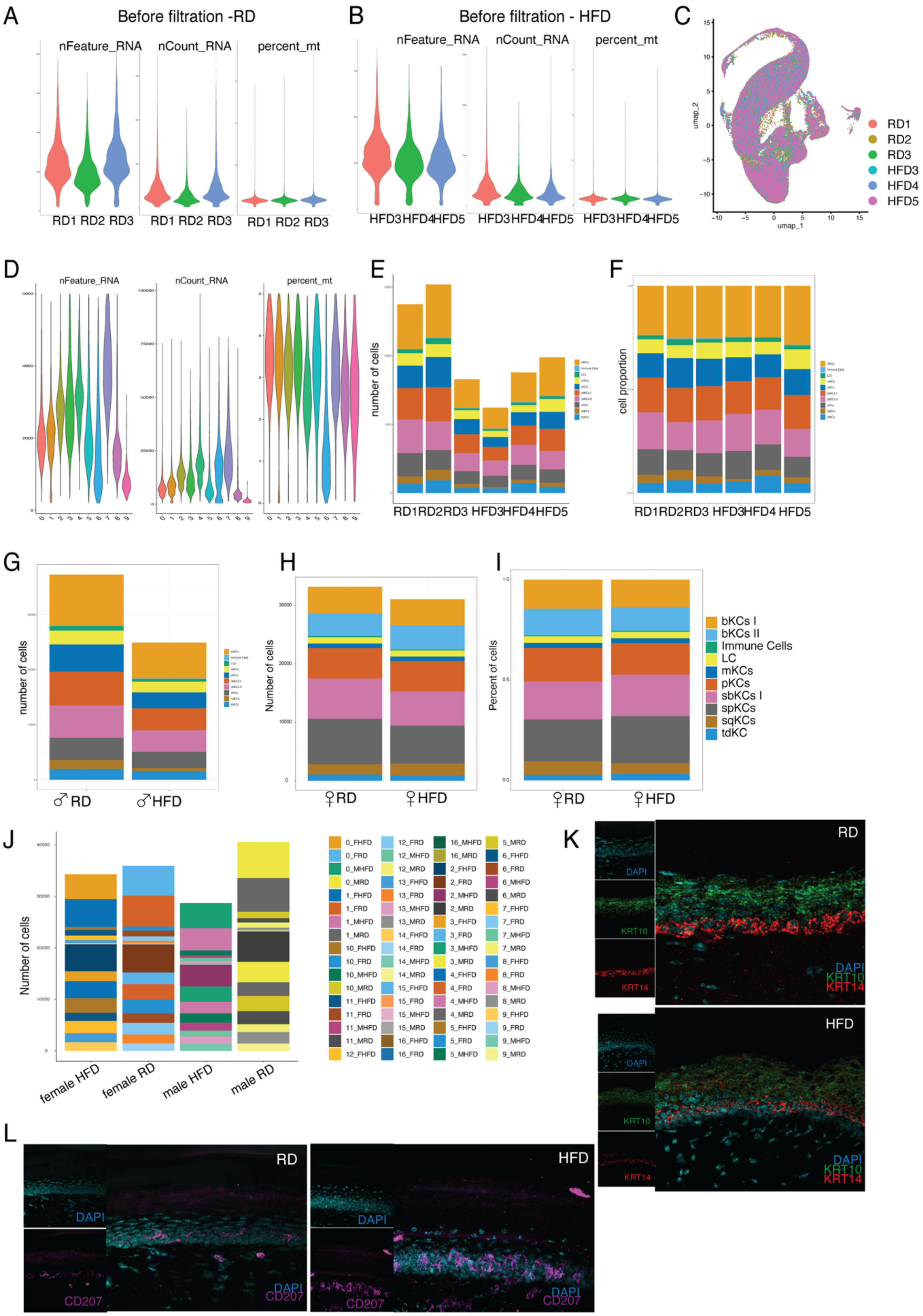
A
Before filtration -RD
nFeature_RNA
nCount_RNA
percent_mt
RD1 RD2 RD3
B
Before filtration - HFD
HFD3 HFD4 HFD5
C
umap_1
umap_2
RD1
RD2
RD3
HFD3
HFD4
HFD5
D
E
number of cells
F
cell proportion
G
number of cells
♂RD
♂HFD
H
Number of cells
♀RD
♀HFD
I
Percent of cells
bKCs I
bKCs II
Immune Cells
LC
mKCs
pKCs
sbKCs I
spKCs
sqKCs
tdKC
J
Number of cells
female HFD
female RD
male HFD
male RD
K
RD
HFD
DAPI
KRT10
KRT14
L
CD207

Supplemental Figure 4. Quality Control (QC) analysis of scRNAseq of RD and HFD paw epidermis (A-B) QC analysis of RD and HFD samples before applying filtration. **(C)** UMAP dimensionality reduction of each sample from RD and HFD show a full overlap **(D)** Cluster QC analysis after filtration for combined RD and HFD. **(E)** Number of cells in each cluster in each sample: RD1=14781, RD2=16221, RD3=9498, HFD3=7521, HFD4=10306, HFD5=11123 cells. **(F)** Cell proportion in each cluster in each sample. **(G)** Total number of cells in combined samples for RD and HFD: RD=40500 and HFD=28950 cells. **(H-I)** QC analysis of female scRNAseq RD and HFD datasets showing the number of cells (J) and the percentage of cells (K) in each cluster. **(J)** Barplots show the number of cells in each cluster in the integration male and female scRNAseq data analysis. **(K)** Representative confocal images of skin sections from RD and HFD paw male mice showing the expression and localization of undifferentiated *Krt14*$^+$ (red) and differentiated *Krt10*$^+$ (green) keratinocytes. **(L)** expression of LCs (*Cd207*/Langerin) in magenta in skin sections from paw male RD and HFD mice.

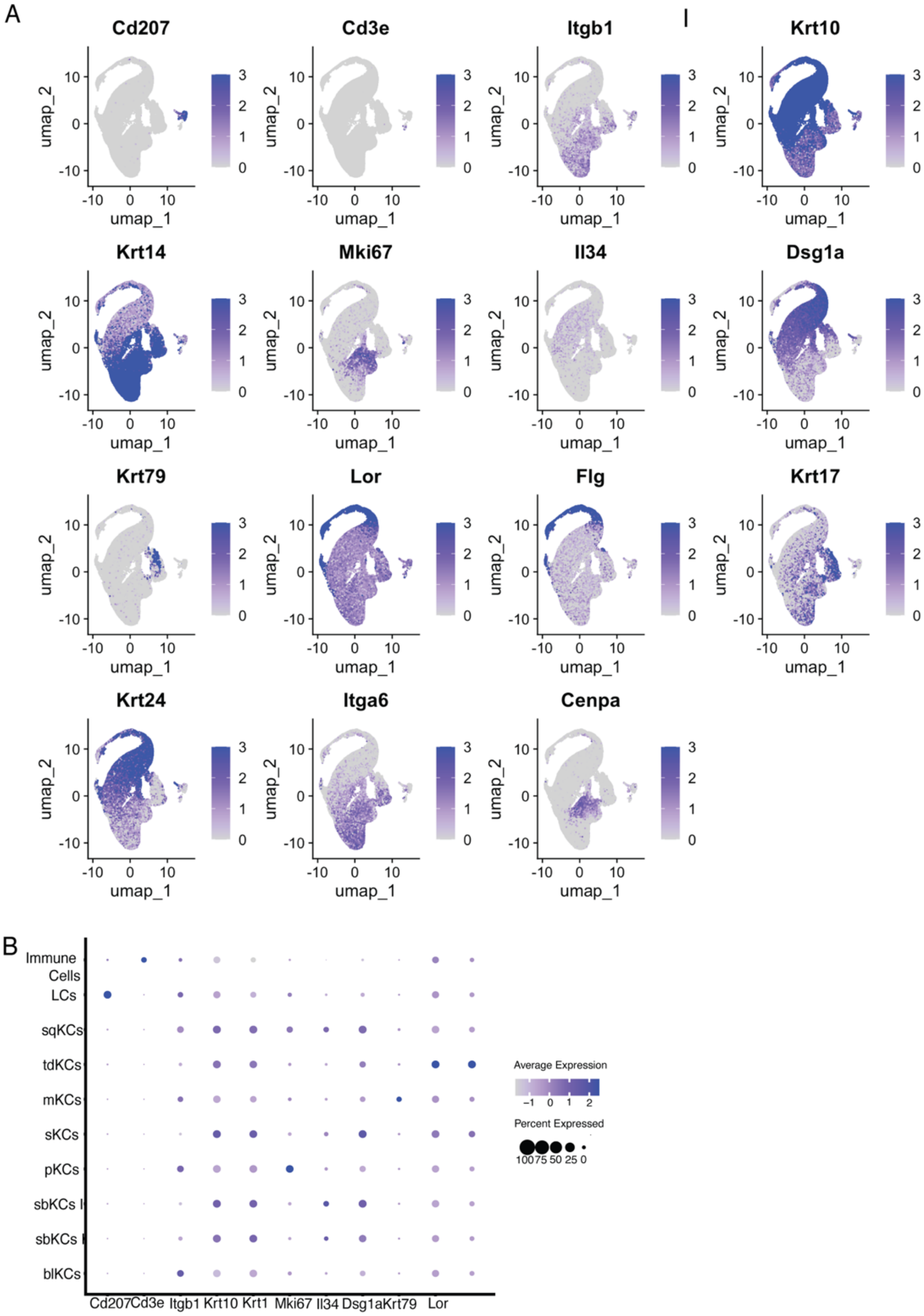

Supplemental Figure 5. Profiling of epidermal cell clusters (A) Feature plots of selected marker genes for cluster identification **(B)** DotPlot shows genes identified as clusters markers in female RD and HFD scRNAseq datasets.

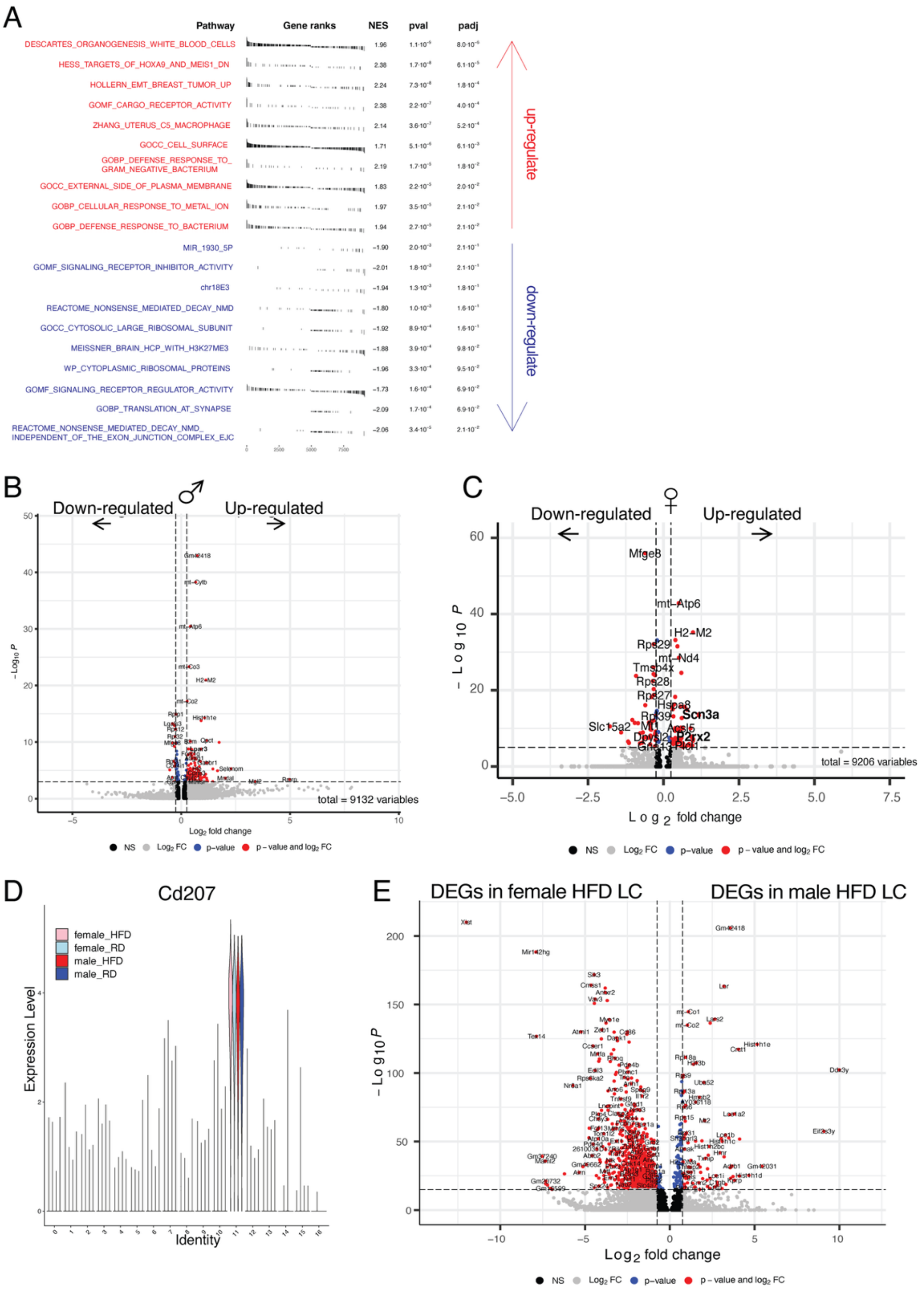

Supplemental Figure 6. DEGs in LCs in HFD male and female mice (A) GSEA table shows top10 upregulated and the top10 downregulated pathways. GSEA based on false discovery rate (FDR) of 0.25. **(B)** Volcano plot of DEGs identified in Langerhans cells cluster in scRNAseq data from mouse paw epidermis. DEGs adjusted for BH correction. Volcano plot generated with EnhancedVolcano package in R. **(C)** Volcano plot of DEGs identified in LCs cluster in scRNAseq data from female mouse paw epidermis. DEGs adjusted for BH correction. **(D)** VlnPlot of *Cd207* expression in epidermal cells in integrated analysis of male and female scRNAseq datasets. **(E)** Volcano plot of DEGs identified in LCs cluster in scRNAseq data from male and female HFD datasets. DEGs adjusted for BH correction. package in R.

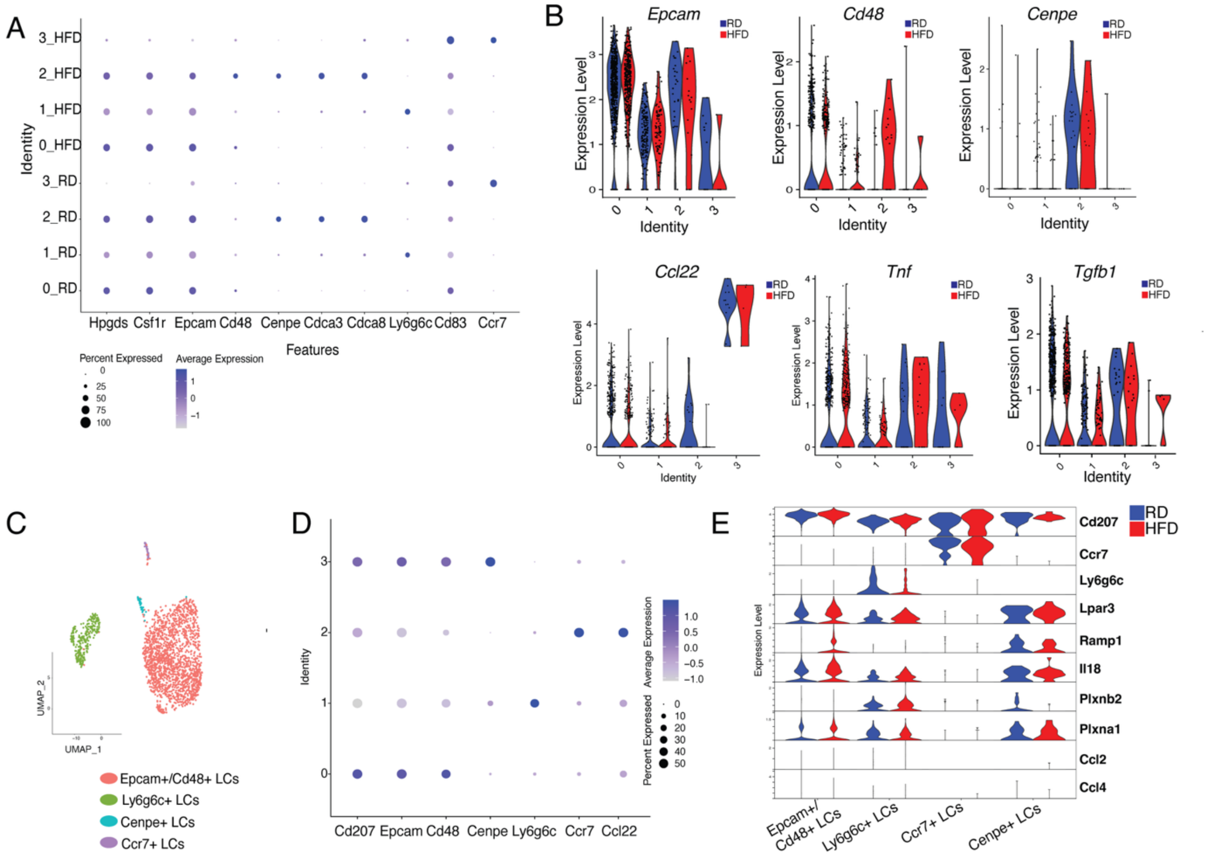

Supplemental Figure 7. DEGs in LCs in HFD male and female mice (A) DotPlot shows the expression of genes adopted for subcluster identification **(B)** Violin plots show the differential expression of genes of interested in LCs subclusters. RD=blue, HFD=red. **(C)** UMAP dimensionality reductions of female LCs subclustering. Four distinct clusters were identified. **(D-E)** Dotplot and stacked violin plot of genes for female LCs subclusters identification and target candidate genes. *Ccl2* was not detected in female LC subclusters.

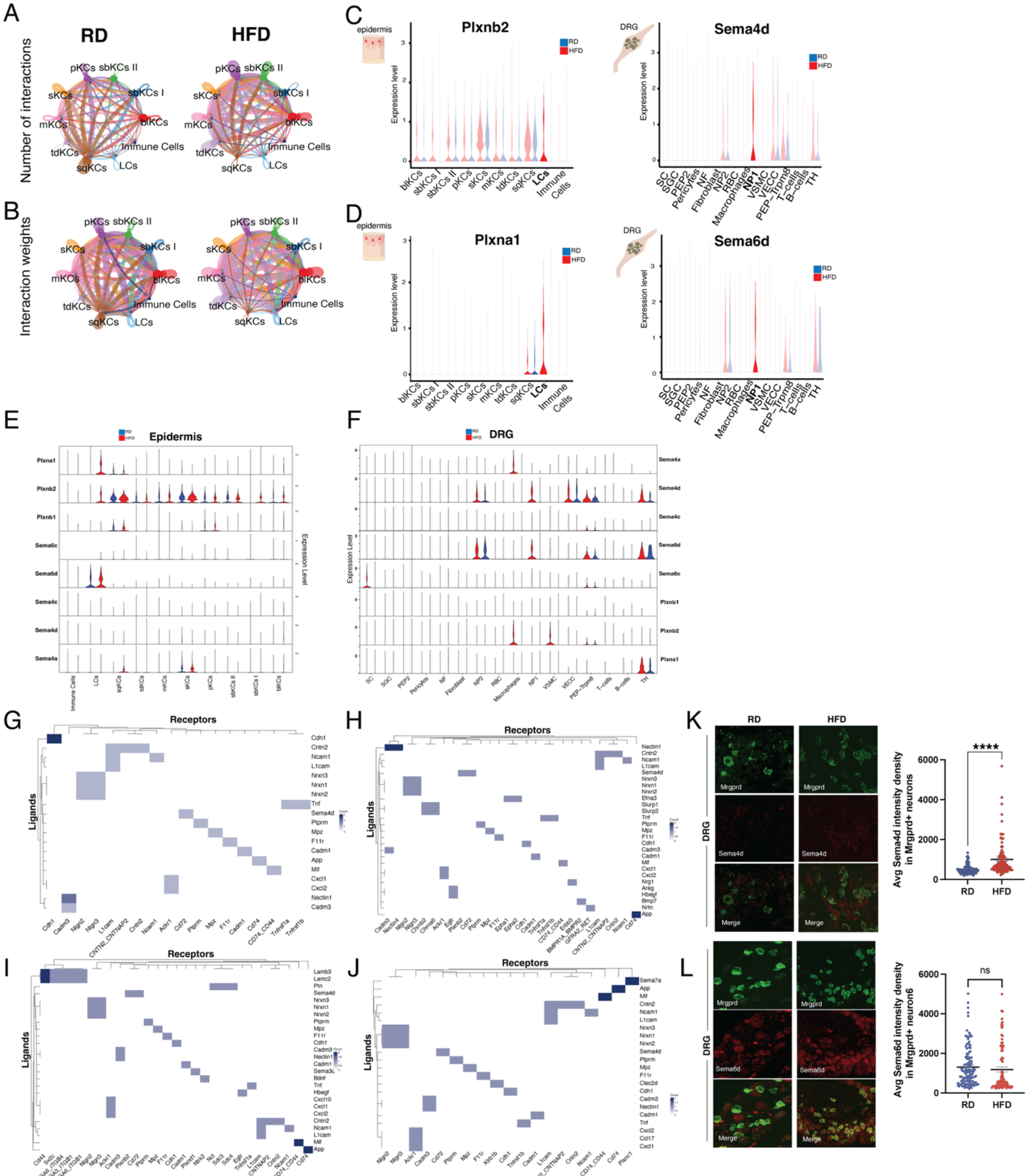

Supplemental Figure 8. Sema-Plxn signaling pathways in RD and HFD male mice (A-B) Circle plots show the number of interactions (b) and the strength of interactions (c) between different cell types in RD/upper panels and HFD/lower panels. Circle sizes are proportional to the size of different cell types and the thickness of the lines indicates a stronger signal. **(C-D)** Violin plots of expression levels of *Plxna1* and *Plxnb2* in the scRNAseq of paw epidermis (g) and Sema4d and Sema6d expression in scRNAseq of DRG (h) of RD and HFD male mice. **(E)** Stacked VlnPlot shows the expression of Sema4 and Sema6 family members and Plxnb2 and Plxna1 family members in RD and HFD epidermis scRNAseq data. **(F)** Stacked VlnPlot shows the expression of *Sema4* and *Sema6* family members and *Plxnb2* and *Plxna1* family members in RD and HFD DRG neurons scRNAseq data. **(G-J)** Heatmaps of inferred signaling pathway

underlying the communication between NP1-positive HFD DRG and each HFD LC subclusters. Analysis performed using Cellchat. **(K)** Representative images of in situ RNAscope of Mrgprd (green) and Sema4d (red) expression in DRG sensory neurons from RD and HFD. Quantification of Average Intensity for each Sema4d dot in Mrgprd+ neurons. 30 dots in Mrgprd+ neurons per animals were quantified. Unpaired two-tailed t-Test p<0.0001 (****). N=3 animals for each diet condition. **(H)** Representative images of *in situ* RNAscope of Mrgprd (green) and Sema6d (red) expression in DRG sensory neurons from RD and HFD. Quantification of Average Intensity for each Sema6d dot in Mrgprd+ neurons. 30 dots in Mrgprd+ neurons per animals were quantified. Unpaired two-tailed t-Test p=0.4277 (ns). RD n=4; HFD n=3.

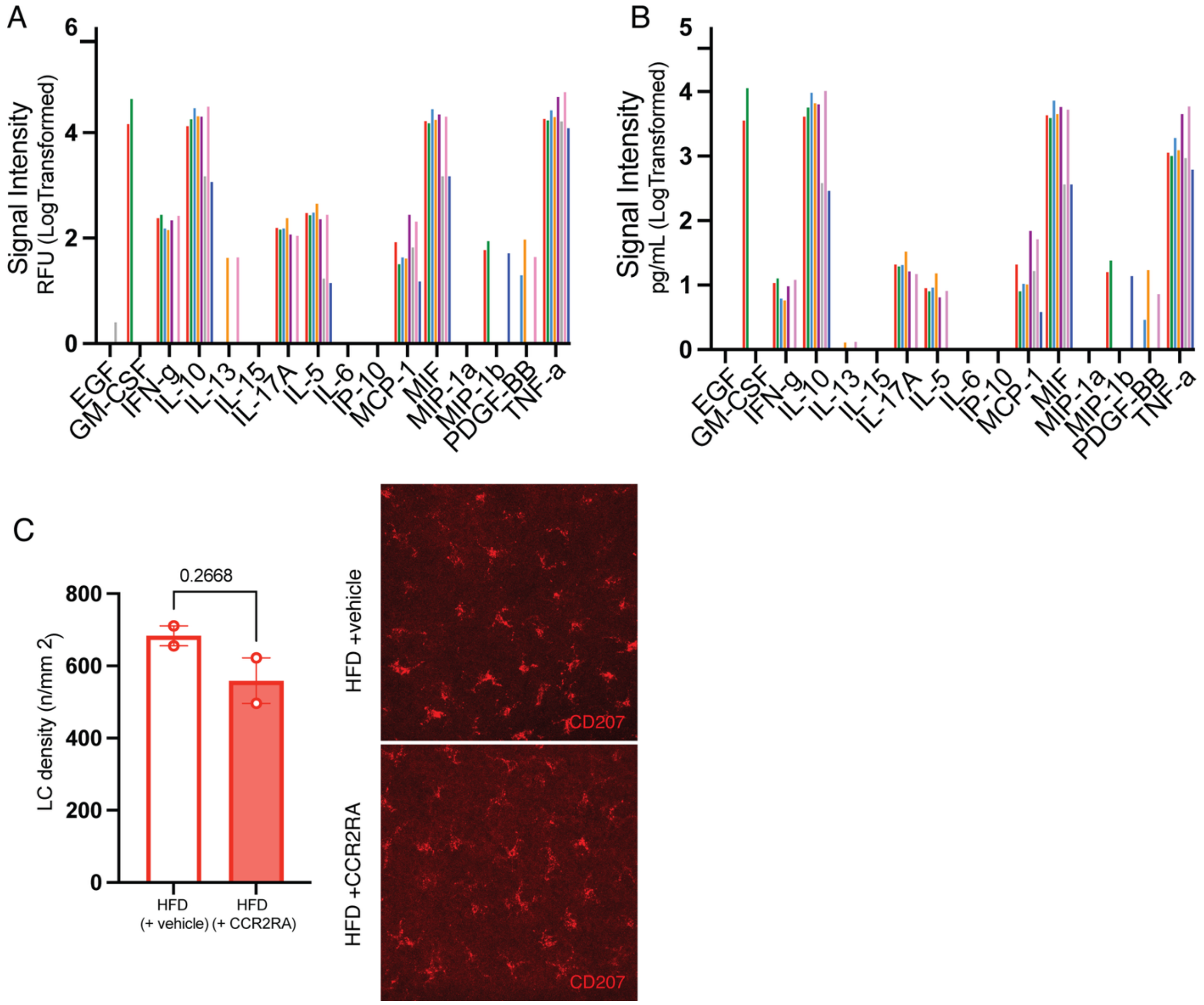

Supplemental Figure 9. (A-B) the high-multiplexing quantitative inflammatory molecule proteomic (CodePlex by Bruker) shows a panel of 16 molecules belonging to the mouse innate immune response and detected in LCs RD and HFD secretome. LCs were stimulated with (1) GM-CSF and the secretome was collected after 12h, (2) TNF-α and the secretome was collected after 12h and 24h and (3) no stimulated and secretome collected after 2h. Molecules are shown as Signal intensity – relative fluorescence unit (RFU and pg/mL, both LogTransformed. **(C)** LC density measured after CCR2RA injection. No differences between HFD vehicle-treated and CCR2RA-treated animals. unpaired t-test with Welch's correction, p = 0.2668. three random areas imaged per animal; n = 2 animals per treatment group

References

1. Menichella DM, Jayaraj ND, Wilson HM, Ren D, Flood K, Wang XQ, et al. Ganglioside GM3 synthase depletion reverses neuropathic pain and small fiber neuropathy in diet-induced diabetic mice. Mol Pain. 2016;12.
2. Jayaraj ND, Bhattacharyya BJ, Belmadani AA, Ren D, Rathwell CA, Hackelberg S, et al. Reducing CXCR4-mediated nociceptor hyperexcitability reverses painful diabetic neuropathy. J Clin Invest. 2018;128(6):2205-25.
3. Zhang S, Edwards TN, Chaudhri VK, Wu J, Cohen JA, Hirai T, et al. Nonpeptidergic neurons suppress mast cells via glutamate to maintain skin homeostasis. Cell. 2021;184(8):2151-66 e16.
4. George DS, Jayaraj ND, Pacifico P, Ren D, Sriram N, Miller RE, et al. The Mas-related G protein-coupled receptor d (Mrgprd) mediates pain hypersensitivity in painful diabetic neuropathy. Pain. 2024;165(5):1154-68.
5. Devigili G, Rinaldo S, Lombardi R, Cazzato D, Marchi M, Salvi E, et al. Diagnostic criteria for small fibre neuropathy in clinical practice and research. Brain. 2019;142(12):3728-36.
6. Koch F, Kampgen E, Schuler G, Romani N. Isolation, enrichment, and culture of murine epidermal langerhans cells. Methods Mol Med. 2001;64:43-62.
7. Lauria G, Bakkers M, Schmitz C, Lombardi R, Penza P, Devigili G, et al. Intraepidermal nerve fiber density at the distal leg: a worldwide normative reference study. J Peripher Nerv Syst. 2010;15(3):202-7.
8. George DS, Jayaraj ND, Pacifico P, Ren D, Sriram N, Miller RE, et al. The Mas-related G protein-coupled receptor d (Mrgprd) mediates pain hypersensitivity in painful diabetic neuropathy. Pain. 2024.
9. Zhang H, Lecker I, Collymore C, Dokova A, Pham MC, Rosen SF, et al. Cage-lid hanging behavior as a translationally relevant measure of pain in mice. Pain. 2021;162(5):1416-25.
10. Hao Y, Stuart T, Kowalski MH, Choudhary S, Hoffman P, Hartman A, et al. Dictionary learning for integrative, multimodal and scalable single-cell analysis. Nat Biotechnol. 2024;42(2):293-304.
11. Jin S, Guerrero-Juarez CF, Zhang L, Chang I, Ramos R, Kuan CH, et al. Inference and analysis of cell-cell communication using CellChat. Nat Commun. 2021;12(1):1088.
12. Wangzhou A, Paige C, Neerukonda SV, Naik DK, Kume M, David ET, et al. A ligand-receptor interactome platform for discovery of pain mechanisms and therapeutic targets. Sci Signal. 2021;14(674).
